# Lanthanide europium MOF nanocomposite as the theranostic nanoplatform for microwave thermo-chemotherapy and fluorescence imaging

**DOI:** 10.1186/s12951-022-01335-7

**Published:** 2022-03-15

**Authors:** Lirong Zhao, Wei Zhang, Qiong Wu, Changhui Fu, Xiangling Ren, Kongpeng Lv, Tengchuang Ma, Xudong Chen, Longfei Tan, Xianwei Meng

**Affiliations:** 1 Laboratory of Controllable Preparation and Application of Nanomaterials, CAS Key Laboratory of Cryogenics, Technical Institute of Physics and Chemistry, Chinese Academy of Sciences, No. 29 East Road Zhongguancun, Beijing, 100190 People’s Republic of China; 2 Department of Nuclear Medicine, Harbin Medical University Cancer Hospital, Nangang District, Harbin, 150086 Heilongjiang People’s Republic of China; 3 Department of Interventional Radiology, Shenzhen People’s Hospital (The Second Clinical Medical College, Jinan University; The First Affiliated Hospital,, Southern University of Science and Technology), Shenzhen, 518020 Guangdong People’s Republic of China; 4 University of Chinese Academy of Sciences, Beijing, 100049 People’s Republic of China

**Keywords:** Lanthanide metal organic frameworks, EuMOF, Microwave thermal therapy, Fluorescence imaging, Theranostic nanoplatform

## Abstract

**Backgrounds:**

Microwave sensitization nanoplatform, integrating multiple functional units for improving tumor selectivity, is of great significance for clinical tumor microwave treatment. Lanthanide europium metal organic framework (EuMOF) is expected to be a theranostic nanoplatform owing to its unique luminescent and microwave sensitization properties. However, it is difficult to be applied to complicated biological systems for EuMOF due to its rapid degradation induced by the solvent molecular and ionic environment. In this work, a luminescent EuMOF nanocomposite (EuMOF@ZIF/AP-PEG, named EZAP) was designed, which brought the multifunctional characteristics of microwave sensitization, fluorescence imaging and drug loading.

**Results:**

Lamellar EuMOF was synthesized by a hydrothermal method. Through the charge adsorption mechanism, the zeolite imidazole framework (ZIF) structure was intensively assembled on the surface of EuMOF to realize the protection. Then, through in-situ Apatinib drug loading and PEG modification, EZAP nanocomposite was finally obtained. Apatinib (AP) was a kind of chemotherapy drug approved by Food and Drug Administration for targeted therapy of tumors. PEG modification increased long-term circulation of EZAP nanocomposite. The physical and chemical structure and properties of EuMOF@ZIF (EZ) were systematically represented, indicating the successful synthesis of the nanocomposite. The toxic and side effects were negligible at a safe dose. The growth of human liver cancer cells and murine liver cancer cells in vitro was significantly inhibited, and the combined microwave-thermal therapy and chemotherapy in vivo achieved high anti-cancer efficacy. Moreover, EZAP nanocomposite possessed bright red fluorescence, which can be applied for tumor imaging in tumor-bearing mice in vivo.

**Conclusion:**

Therefore, EZAP nanocomposite showed high microwave sensitization, excellent fluorescence properties and outstanding drug loading capacity, establishing a promising theranostic nanoplatform for tumor therapy and fluorescence imaging. This work proposes a unique strategy to design for the first time a multifunctional nanoplatform with lanthanide metal organic frameworks for biological applications in tumor therapy and diagnosis.

**Graphical Abstract:**

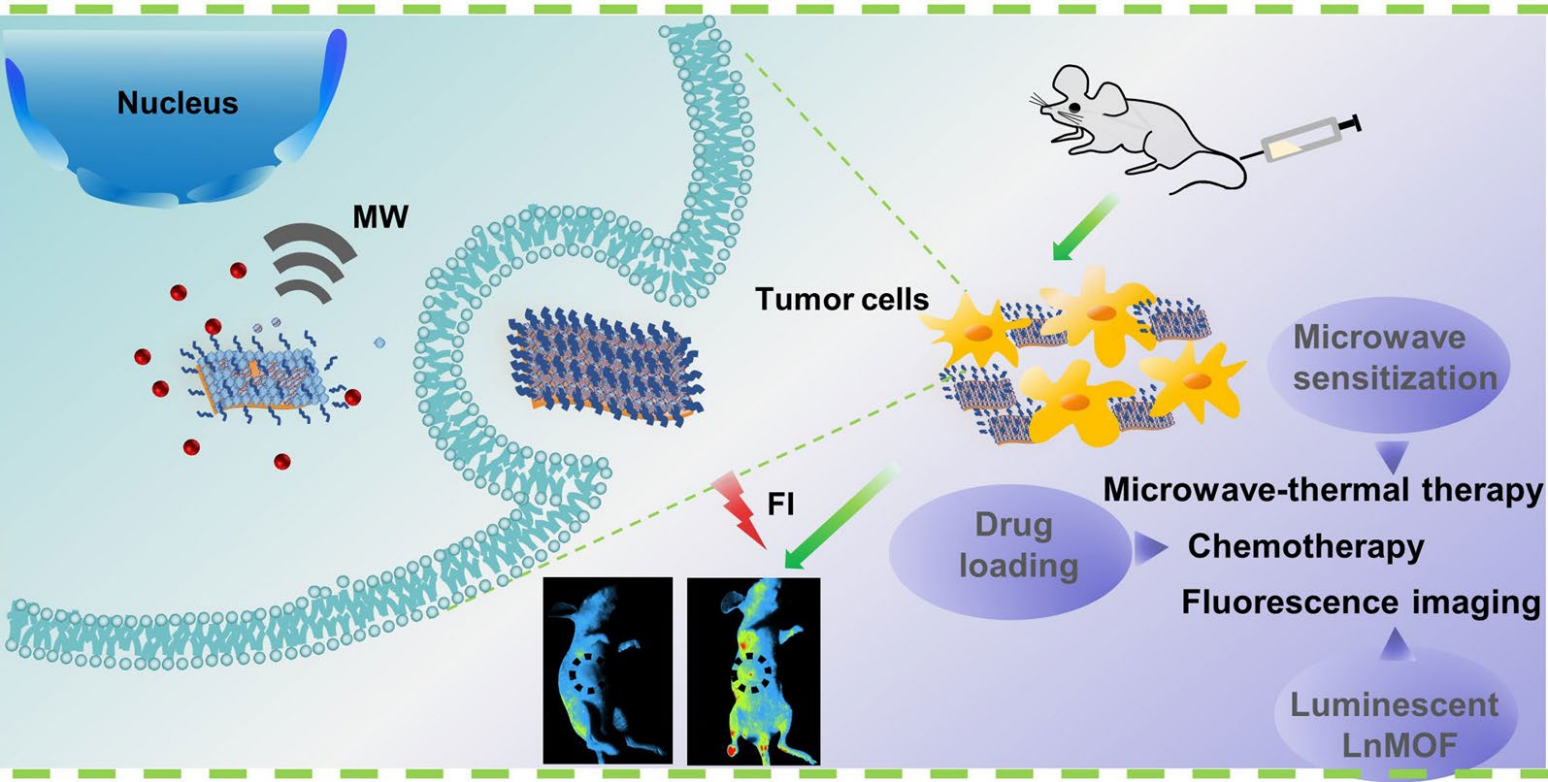

**Supplementary Information:**

The online version contains supplementary material available at 10.1186/s12951-022-01335-7.

## Introduction

Microwave thermal therapy, as a new type of localized cancer treatment for tumor thermal ablation, takes the advantages of minimally invasion and deep penetration [1, 2]. Microwave thermal therapy has been widely used in the clinical treatment of tumors, and is currently the first-line treatment for hepatocellular carcinoma [3–7]. However, microwave thermal therapy is not selective for tumor cells and does not specifically damage to them [8–10]. Thus tumor cells are hardly ablated completely, there will be a high recurrence rate which seriously restricts its clinical application. It also has obvious cytotoxic effect on surrounding healthy tissue. Hence, a variety of microwave sensitizing nanomaterials have been developed [11–14], such as organic materials (gelatin, sodium alginate microcapsules) [15], inorganic materials [16–18] (zirconium dioxide hollow nanospheres [19], carbon nanotubes [20–22]), organic–inorganic hybrid materials (metal organic framework [23–25]). Amongst, synthesis methods and the targeting ability of the organic materials need to be improved [26, 27]. The inorganic nanomaterials are unable to be metabolized and difficult to be eliminated from the body, which limits their clinical applications [28–30]. Metal organic framework materials, as organic and inorganic hybrid materials, are widely used in tumor treatment due to their low toxicity and good biodegradability [31–33]. Most recently, MOFs have high porosity and large specific surface area of interconnected network structure, strong scattering, reflection and dissipation ability, resulting in effective electromagnetic response, becoming an alternative microwave sensitizer [34].

As a luminescent material, lanthanide metal organic frameworks (LnMOFs) have shown attractive application prospects in the fields of chemical sensing, temperature sensing, biological detection and biological imaging [35, 36]. In particularly, abundant electron energy levels and a large number of energy level transitions in the electron configuration endow the lanthanide europium metal organic frameworks with the most potential microwave sensitization agents [37–39]. The additional vital part of Eu metal organic framework is the specific fluorescence, high quantum efficiency, long fluorescence lifetime and characteristic emission peak position, as imaging agent plays the important role for tumor diagnosis and treatment. Therefore, EuMOF is a promising candidate for efficacy theranostic agent to integrating the high microwave sensitizing and fluorescent imaging [40, 41].

However, the research of EuMOF in nanomedicine field has hardly been carried out and reported, especially for combined treatment of tumors. Because EuMOF was easily degraded by the surrounding ionic environment and solvent molecules, resulting in fluorescence quenching [42]. Until now, the EuMOF theranostic nanoplatform has not been achieved for that [43].

**Scheme 1:**
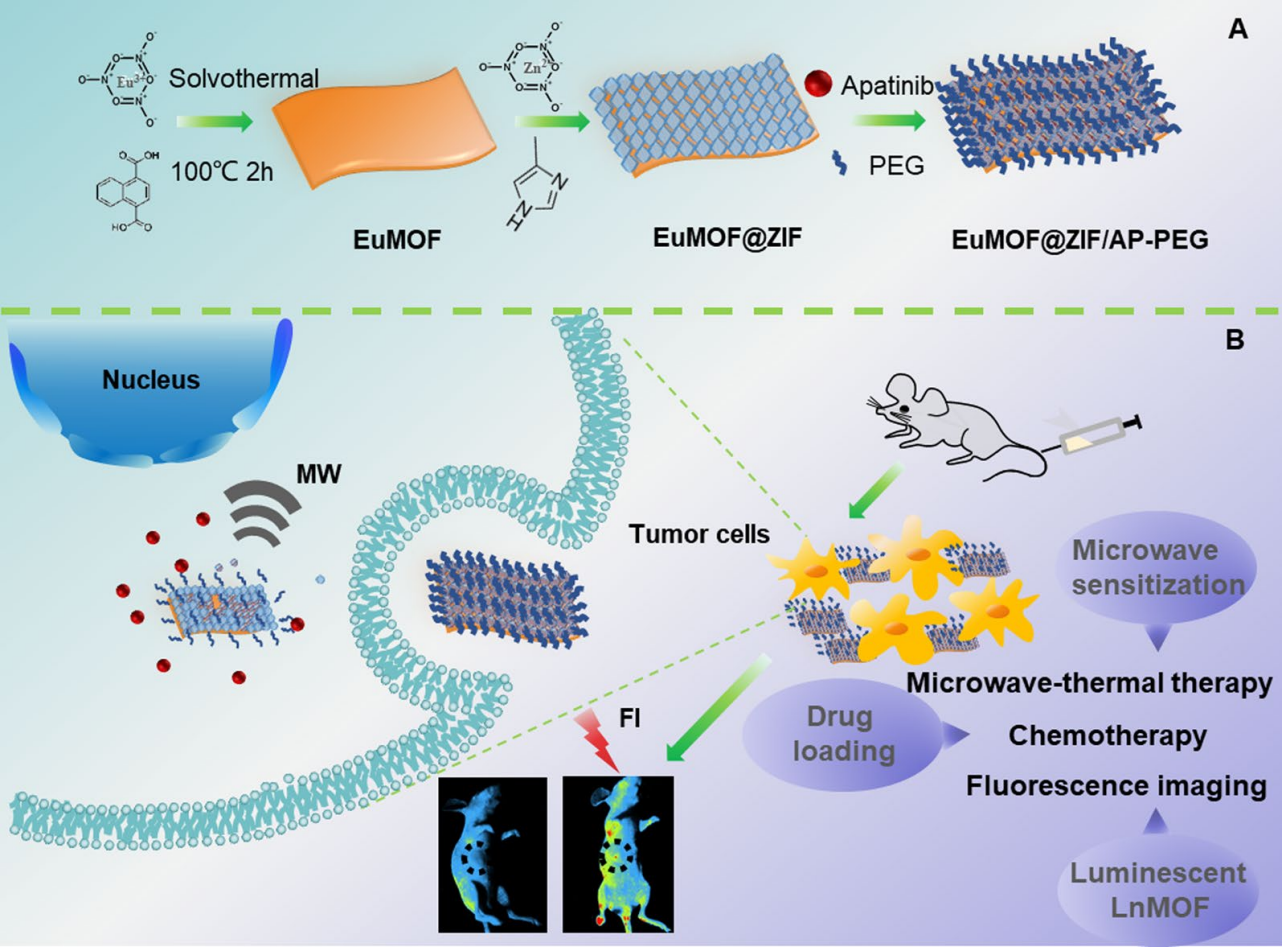
EZAP formulation process and biological application. **A** The synthesis of lanthanide MOF nanocomposite and **B** its application in microwave thermal-chemotherapy of tumor and in vivo fluorescence imaging

Here, to solve this problem, a stable zeolite imidazole framework structure was introduced on the surface of EuMOF to provide coating protection for EuMOF, while as an excellent drug loading system due to its porosity and high specific surface [44–46]. Therefore, we reported a luminescent EuMOF nanocomposite (EuMOF@ZIF/AP-PEG, named EZAP) as a multifunctional nanoplatform for microwave-thermal therapy and chemotherapy and fluorescence imaging of tumors (Scheme 1). EuMOF was synthesized by solvothermal method. After ZIF growth on the surface of EuMOF, in-situ Apatinib loading and PEG modification, EZAP nanocomposite was finally obtained [47]. EZAP nanocomposite showed high microwave sensitization, excellent fluorescence properties and good drug loading capacity. The physical and chemical structure and properties of EZ, drug loading in vitro and tumor fluorescence imaging in vivo and in vitro were systematically studied. EZAP entered the tumor site through EPR effect, improving the therapeutic effect of microwave thermo-chemotherapy as a microwave sensitizer, and observing the tumor as a fluorescent probe. The anti-tumor effect of EZAP nanocomposite under microwave irradiation in vivo and in vitro was studied in detail. The tumor fluorescence imaging was evaluated to prove the potential of EuMOF@ZIF as a fluorescent nanoprobe. In this multifunctional platform, we have not only created a new microwave sensitizer material, but also innovatively pushed lanthanide MOF to the the field of microwave responsive nanomedical science in vivo, including in vivo tumor imaging and treatment. It also provides a novel idea for the biological application of lanthanide MOFs.

## Methods

### Materials

Europium nitrate hexahydrate, 1, 4-naphthalene dicarboxylic acid, cetyl trimethyl ammonium bromide, zinc nitrate hexahydrate, 2-methylimidazole, triethanolamine, polyvinylpyrrolidone, n, n-dimethylformamide, absolute ethanol, absolute methanol, all reagents are analytically pure.

### Synthesis of EuMOF

Nano-scale EuMOF was synthesized using europium nitrate hexahydrate [Eu(NO_3_)_3_·6H_2_O] and 1, 4-naphthalene dicarboxylic acid (1,4-H_2_NDC) as raw materials. The metal nitrate Eu(NO_3_)_3_·6H_2_O (1.25 mmol) and the organic ligand 1,4-H_2_NDC (1.25 mmol) were added into the solvent DMF (100 mL) in equal proportion. After that, surfactant CTAB and pH regulator TEOA(NCTAB:NTEOA = 1:2) were added, as well as dispersant PVP (100 mg). After stirring and mixing evenly, the mixed solution is added to the appropriate size of the reaction kettle. After reaction for 2 h in the oven at 100 ℃, the solution was centrifuged 10000 rpm for 5 min and washed with ethanol and water for several times. White product of EuMOF was obtained by freeze-drying produces.

### Synthesis and characterization of EuMOF@ZIF

EuMOF (40 mg) was dispersed in ethanol (4 mL), and PVP (80 mg) was dissolved in ethanol (1 mL). PVP alcohol solution was added drop by drop into EuMOF solution, stirred by magnetic force at room temperature for 4 h, centrifuged at 10,000 rpm for 5 min, washed with anhydrous ethanol for 3 times, and freeze-dried to obtain white product. Weighed 20 mg of the above products and dispersed them in 10 mL methanol, added 1 mL of Zn(NO_3_)_3_·6H_2_O (0.27 mol/mL) methanol solution, stirred for 10 min, added 3 mL of 2-MI(0.65 mol/mL) methanol solution, stirred at room temperature for 6 h. The reaction was centrifuged at 8000 rpm for 5 min, washed with anhydrous ethanol for 3 times, and freeze-dried to obtain a white product the EuMOF@ZIF nanocomposite. Scanning electron microscopy (S-4800, Hitachi) and biological transmission electron microscopy (HT7700, Hitachi) were used to observe the morphology of the EuMOF@ZIF nanocomposite. The elemental distribution and content of the samples were determined by high resolution transmission electron microscopy (JEM-2100F, Hitachi). The hydrodynamic diameter and potential of the sample were measured by the zetasizer (Nano ZSE, Malvern). X-ray diffractive analysis (D8 focus, Bruker) of the sample was performed with CuKα light source at a scanning rate of 0.02 deg/s. The infrared absorption curves of the samples were determined by the Fourier infrared absorption (Excalibur 3100, Varian) analyzer. The UV–vis absorption spectra of the samples were measured by UV–vis spectrophotometer (Cary5000, Agilent).

### In vitro microwave thermal performance

The prepared EuMOF@ZIF nanocomposite was tested by microwave irradiation to study its microwave heating effect. Firstly, EuMOF@ZIF nanomaterials with a concentration gradient of 0, 2, 4, 8 mg/mL were prepared by using 0.9% NaCl as the solvent. One mL of the samples was put into the sample tank of the microwave instrument, and the microwave irradiation was carried out at a frequency of 1.8 W for 5 min. The temperature data were recorded every 10 s. During this process, the red-heat external imager was used to monitor the temperature changes in real time.

### In vitro luminescent properties

The fluorescence test of the prepared EuMOF@ZIF nanocomposite proves its excellent properties. The best excitation wavelength of the nanomaterial is 280 nm, and the maximum emission peak is 618 nm. Firstly, under the condition of certain material concentration, the fluorescence curves of the EuMOF@ZIF nanocomposite at different excitation wavelengths were obtained, and then the emission spectra of materials with different concentrations (5, 4, 2, 1, 0.5, 0.25, 0.125, 0.0625, 0.03125 mg/mL) at the optimal excitation wavelengths were explored.

### In vitro drug loading and release behavior

In order to study the treatment in animals, PEG modification was carried out to improve biocompatibility. 10 mg EuMOF@ZIF nanocomposite was dispersed in 3 mL ethanol, and the same volume of SH-PEG (10 mg) ethanol solution was added, stirring at room temperature for 2 h. After centrifuged at 8000 rpm for 5 min and washed with ethanol for 3 times, white product EuMOF@ZIF-PEG nanocomposite was obtained by freeze-dried for later use. The preparation of EuMOF@ZIF/AP-PEG nanocomposite is similar to the drug loading process, and the material is prepared by in-situ loading with a mass ratio of 1.0 to Apatinib. The specific operations are as follows: EuMOF@ZIF nanocomposite 10 mg were dispersed in 3 mL ethanol and 1 mL of Apatinib solution (10 mg/mL) was prepared. The two solutions were mixed and stirred evenly, then 1 mL of SH-PEG (10 mg/mL) was added and stirred at room temperature for 4 h. The solution was centrifuged and washed three times with ethanol. The EuMOF@ZIF/AP-PEG nanocomposite containing chemotherapeutic drugs was obtained. The supernatant was collected and diluted to an appropriate volume at a certain multiple. The UV–vis absorption of Apatinib was detected and the loading rate was calculated. The standard concentration-absorption curve of Apatinib was Abs = 0.02057C + 0.00659 (R^2^ = 0.9999) according to our group's previous report [48]. The precipitates were modified with PEG and drug release experiments were carried out under neutral physiological conditions. The standard curve of drug release was drawn, and the amount of drug release was calculated according to the absorbance value of drug release.

### Cytotoxicity

The biosafety of EZ nanocomposite was evaluated after PEG modification. HepG2 cells were incubated with 5% CO_2_ in the incubator at 37 ℃. When the density of HepG2 cells grew to 70–80%, the old medium was sucked out, washed by PBS for 3 times, and then trypsin was added to digest HepG2 cells. The trypsin was sucked out, the newly prepared medium was added, the digested cells were gently blown and placed in a 96-well plate at a density of 7 × 10^3^ cells per well for 24 h. Then different concentrations (0, 12.5, 25, 50, 100, 200 μg/mL) of the EuMOF@ZIF-PEG nanocomposite was co-cultured with cells for 24 h, and then MTT (0.5 mg/mL) was added into the culture system. After the MTT was removed, dimethyl sulfoxide was added. The absorbance (absorption peak 492 nm) of each plate on the 96-well plate was measured using a microplate analyzer to calculate the cell viability and evaluate the biosafety.

### VEGF immunofluorescence test

HepG2 cells were used as cell model to evaluate the inhibitory effect of EuMOF@ZIF/AP-PEG on vascular endothelial growth factor expression. Tumor cells were cultured into 6-well plate (approximately 1 × 10^5^ cells per well) at 37 ℃, 5% CO_2_ for 18 h. The experiment was divided into four groups: control, Apatinib (20 μg/mL), EZAP (100 μg/mL), EZP (100 μg/mL). After incubating with each group of cells for 24 h, the old medium was sucked out. Cells were fixed with immunofluorescence staining fixative, washed 2–3 times with cleaning solution, sealed with blocking solution, incubated with VEGF antibody for 12 h, washed 2–3 times, and incubated with green-fluorescent anti-VEGF antibody 1 h. The cells were washed 2–3 times and stained with DAPI with blue fluorescence for 3 min. Finally, the cells were washed 2–3 times and the immunofluorescence of the cells was observed under a fluorescence microscope.

### In vitro microwave thermo-chemotherapy evaluation

To evaluate the microwave thermal-chemotherapy performance of nanocomposite at the cell level, tumor cell inhibition experiments were carried out. HepG2 cells were evenly seeded into 6-well plates at a density of 1 × 10^5^ cells per well and cultured until cells adhered to the wall. The cells were divided into seven groups and treated with different treatment methods: (1) control group, (2) free Apatinib group, (3) EuMOF@ZIF-PEG group, (4) EuMOF@ZIF/AP-PEG group, (5) MW group, (6) EuMOF@ZIF-PEG + MW group, (7) EuMOF@ZIF/AP-PEG + MW group. The details are as follows: new medium was added to the groups 1 and 5; equivalent medium (Apatinib concentration was 20 μg/mL) was added in the group 2. The group 3 and group 6 were added with equivalent medium (EuMOF@ZIF-PEG concentration was 100 μg/mL); Groups 4 and group 7 were added with equivalent culture medium (EuMOF@ZIF/AP-PEG concentration was 100 μg/mL) for 24 h, then the cells were digested with trypsin, centrifuged, the culture medium was removed. 1 mL of culture medium was added, and the cells of each group were dispersed evenly. Free Apatinib group, EuMOF@ZIF-PEG group, EuMOF@ZIF/AP-PEG group were transferred to 96-well plate at 100 μL/well (approximately 6 × 10^3^ cells per well). The other three groups were MW group, EuMOF@ZIF-PEG + MW group, EuMOF@ZIF/AP-PEG + MW group were digested, centrifuged, and the medium was removed. DMEM was gently blown and dispersed, irradiated with MW (1.8 W) for 5 min. DMEM was poured after centrifugation. One mL medium was added, and then added into 96-well plate at 100 μL per well (approximately 6 × 10^3^ cells per well) for 24 h. MTT assay was performed to calculate cell viability and evaluate antitumor efficiency.

In order to further verify the tumor inhibition effect under microwave irradiation, living-dead cell staining experiment was carried out. After each group of cells had been treated, they were inoculated into a six-well plate (approximately 1 × 10^5^ cells per well). The green fluorescent calcium xanthocyanin solution and red fluorescent propidium iodide solution were added to the medium under dark condition, respectively. The tumor cells were incubated with dyes for 10 min. Then, the cell staining was observed under a fluorescent confocal microscope to obtain the cell death status and evaluate the inhibition effect of tumor cells.

### In vivo microwave thermo-chemotherapy

Female BALB/c mice (16–18 g) were used as animal model. After hair removal, H22 tumor cells (100 μL, 2 × 10^6^) were inoculated into the armpits of mice. When the tumor grows to the suitable size (130 ± 30 mm^3^), tumor-bearing mice were randomly divided into six groups with three mice in each group: control group (without any treatment), EuMOF@ZIF/AP-PEG group (containing drug material, tail i.v., 50 mg/kg), free Apatinib group (tail i.v.,10 mg/kg), MW group (1.8 W, 5 min), EuMOF@ZIF-PEG + MW group (without drug material plus microwave, tail i.v., 50 mg/kg, 1.8 W, 5 min), EuMOF@ZIF/AP-PEG + MW group (drug containing materials plus microwave, tail i.v., 50 mg/kg, 1.8 W, 5 min). During this period, the temperature changes of mice tumors during microwave treatment were monitored in real time by using the infrared thermal imager. Daily changes in body weight, tumor volume and survival of each mouse in each group were recorded, and photos were taken for preservation. Mouse tumor volume was calculated according to the following formula: Mouse tumor volume = (length × width^2^)/2, the unit was mm^3^. After 14 consecutive days of observation, curves were drawn according to the tumor volume of mice to evaluate the therapeutic effect of EuMOF@ZIF/AP-PEG nanocomposite on liver cancer in vivo.

### In vivo fluorescence imaging

In addition, fluorescence imaging signals of tumor-bearing mice were collected at different time points using the Fusion FX7 Spectra multifunctional imaging system. All animal experiments were carried out in accordance with the agreement of Harbin Medical University Cancer Hospital (No: SYXK2019-001; SCXK2019-001).

### Histology analysis

After 14 days, the main tissues of mice (heart, liver, spleen, lung and kidney tumors) were collected for histological analysis to further evaluate the toxicity of EuMOF@ZIF/AP-PEG nanocomposite on tumor-bearing mice and the efficacy of tumor treatment. The organs were immersed and fixed in 4% neutral formaldehyde for 14 days. After dehydration, transparency and paraffin dipping, the tissue fragments were embedded in paraffin. After solidification, the organs were cut into slices of 5 μm and fixed on the slides for drying. After H&E staining and drying in the fume hood, the tissues were examined using a microscope.

### Acute toxicity experiment

In vivo acute toxicity tests were used to further evaluate the biosafety of multifunctional EZAP nanocomposite. Acute toxicity test was performed in ICR mice in vivo. Mice were randomly divided into three groups (control group, 50 mg kg^−1^ and 75 mg kg^−1^ EZAP). Then, EZAP with different concentrations was injected into the experimental mice through the tail vein. The control mice were observed for 14 days without any treatment. Routine blood analysis was performed with mouse blood.

## Results and discussion

### Fabrication and characterization of EuMOF@ZIF

**Fig. 1:**
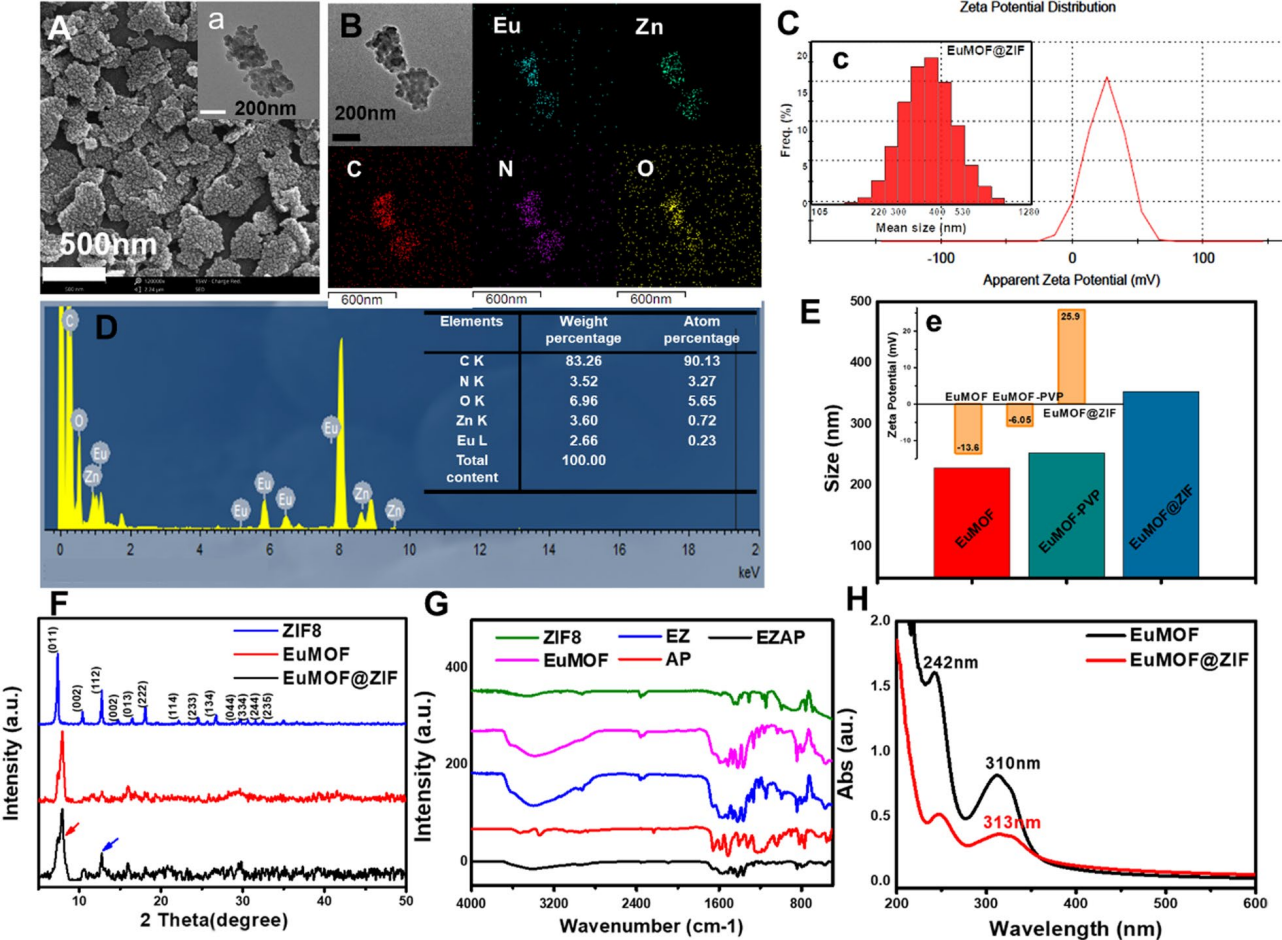
Characterization of EuMOF@ZIF nanocomposite. **A** SEM image of EuMOF@ZIF, TEM image of EuMOF@ZIF in the inset. **B** Elemental mapping of EuMOF@ZIF. **C** Zeta-potential of EuMOF@ZIF, particle size distribution of dynamic light scattering of EuMOF@ZIF in the inset. **D** EDS of EuMOF@ZIF, element content of EuMOF@ZIF in the inset. **E** Particle size histogram of EuMOF, EuMOF-PVP, EuMOF@ZIF, zeta-potentials of EuMOF, EuMOF-PVP, EuMOF@ZIF in the inset. **F** XRD, **G** FTIR of ZIF8, EuMOF, EuMOF@ZIF. **H** UV–vis absorption of EuMOF, EuMOF@ZIF

The realization of multifunctional nanoplatforms depends on rational design of materials and successful characterization of structures. First of all, a lamellar EuMOF was prepared by solvothermal self-assembly of lanthanide europium metal ions and organic ligands of 1, 4-naphthalene dicarboxylate according to the synthesis conditions of MOF (Additional file 1: Figure S1). A metal organic framework of zeolite imidazole was used for coating protection. Zn^2+^ was adsorbed onto the surface of EuMOF by electrostatic charge adsorption process. Then, through coordination between 2-MI ligand and Zn^2+^ ion, they reacted on the surface of EuMOF to grow ZIF, and finally synthesized the EuMOF@ZIF nanocomposite. Transmission electron microscopy (TEM) and Scanning electron microscopy (SEM) revealed that the particle size of EuMOF@ZIF nanocomposite was about 365 nm, and the morphology of EuMOF@ZIF nanocomposite showed the appearance of rice crust (Fig. 1A). The elemental mapping showed that Eu, Zn, C, N and O were uniformly distributed in lamellar materials (Fig. 1B). Dynamic light scattering tests showed that the hydrodynamic diameter of EuMOF, EuMOF-PVP and EuMOF@ZIF were about 228 nm, 253 nm and 353 nm, respectively (Additional file 1: Figures S2, S4, Fig. 1C). Zeta potential test data showed that the potential of EuMOF, EuMOF-PVP and EuMOF@ZIF were − 13.6 mV, − 6.05 mV and + 25.9 mV, respectively (Additional file 1: Figures S3, S5, Fig. 1E). The X-ray diffraction showed that the EuMOF@ZIF nanocomposite had the same diffraction peak position as ZIF, such as 7θ, 11θ, 13θ, 18θ have similar peak position, compared with the X-ray diffraction pattern of EuMOF given in the literature, obtained similar results (Fig. 1F). To further confirm the functional groups contained in the EuMOF@ZIF nanocomposite, infrared absorption test was carried out. The ligands of EuMOF contain naphthalene rings, and the characteristic skeleton vibrations of the naphthalene rings are around 1418 and 1458 cm^−1^. O–H, N–H stretching vibration zone in 3750–3000 cm^−1^; the stretching vibration zone of carbonyl group is 1900–1650 cm^−1^. FTIR showed that the EuMOF@ZIF nanocomposite had the same chemical bonds and groups as ZIF, and the spectral lines of EuMOF and EuMOF@ZIF were basically the same (Fig. 1G). The existence of Eu, Zn, C, N and O elements was confirmed by EDS test. At the same time, the table listed the weight and atomic percentage of each element. The weight percentage of Eu and Zn was 2.66% and 3.60% respectively, indicating that the content of Zn element was slightly higher than that of Eu element, which may be due to the dense coating of ZIF on the surface of EuMOF (Fig. 1D). The UV–vis absorption spectra showed that the absorption of EuMOF was in the band of 200–600 nm, and there were significant maximum absorption peaks at 242 nm and 310 nm. Compared with EuMOF, the maximum absorption peak of EuMOF@ZIF nanocomposite had a slight red shift, but the peak pattern was basically similar (Fig. 1H). We have performed stability tests for EuMOF@ZIF and EuMOF, placing EuMOF@ZIF in a PBS solution containing ions and solvent molecules and observing its morphologies at different time points. The lamellar structure of EuMOF collapsed at 0.5 h, and the original structure could not be seen with the extension of time. However, at 0.5 h, the rice crust structure of EuMOF@ZIF remained, and ZIF pellets fell off with the extension of time, but the lamellar structure of EuMOF was still clearly visible. The experimental results showed that the EuMOF@ZIF had certain stability in neutral PBS and was consistent with the SEM, the introduction of ZIF did increase the stability of EuMOF (Additional file 1: Figure S6). The stability of EuMOF@ZIF nanocomposite was also tested by sedimentation experiments in PBS solutions at different pH values, such as 7.4, 5.7 (Additional file 1: Figure S7). EuMOF in PBS solution at pH 7.4 had obviously fast settlement after 2 h, while EuMOF@ZIF nanocomposite was quite stable in PBS solution at pH 7.4. The results of these characterization tests indicated that the synthesis of nanocomposites is successful.

### The evaluation of microwave thermal property

**Fig. 2:**
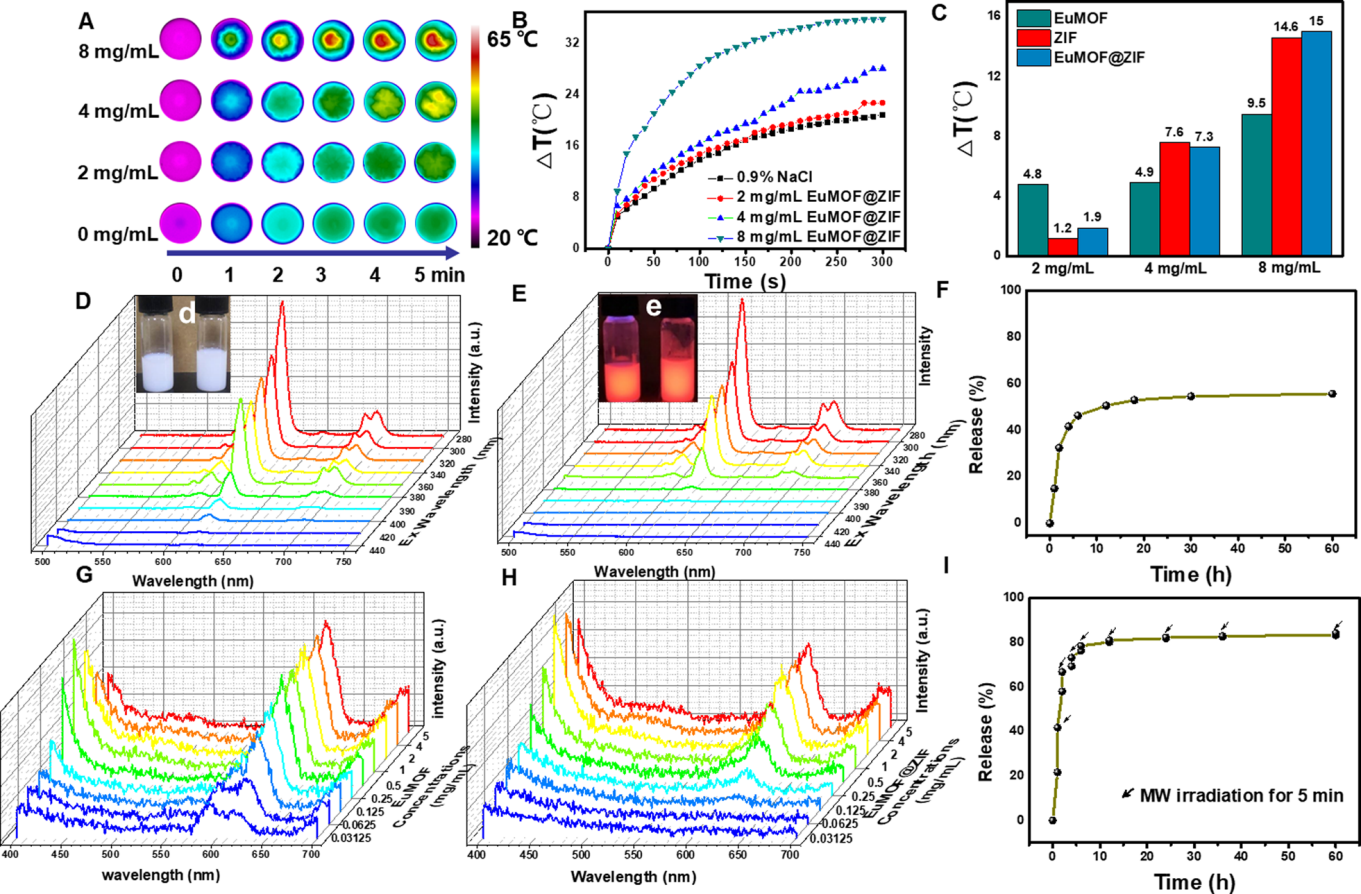
Microwave thermal property, luminescent property and drug loading and release of EuMOF@ZIF nanocomposite. **A** Infrared thermal images, **B** temperature variation curves, of EuMOF@ZIF (0, 2, 4, 8 mg/mL) under microwave irradiation for 5 min. **C** Histogram of temperature rise between EuMOF@ZIF (2, 4, 8 mg/mL) and blank concentration group, as well as EuMOF, ZIF. Emission spectra of **D** EuMOF, **E** EuMOF@ZIF at different excitation wavelengths, pictures in the inset from left to right of EuMOF, EuMOF@ZIF. **d** In daylight. **e** Under ultraviolet light. **F** Drug release rate of EZ loaded Apatinib under neutral physiological condition. **I** Drug release rate of EZ loaded Apatinib under microwave stimulation (microwave irradiation for 5 min, 1.8 W). Emission spectra of **G** EuMOF, **H** EuMOF@ZIF at different concentrations

Commonly, the mechanism of microwave thermal sensitization is to convert microwave energy into the energy of intense collision motion through the physical action of electromagnetic response, and then convert it into heat by means of the dissipation action of this motion. Nanoscale MOFs and their derivatives, with its high porosity and large specific surface area, produce strong multiple scattering and reflection, and effectively improve the electromagnetic response, which have been proved to be a new generation of microwave sensitization agent [34]. In order to evaluate the microwave thermal sensitization performance of the EuMOF@ZIF nanocomposite, temperature test and infrared thermal imaging real-time monitoring were carried out under microwave irradiation. The EuMOF@ZIF nanocomposite dispersed in saline was prepared with different concentrations (0, 2, 4, 8 mg/mL) and irradiated by MW for 5 min (1.8 W cm^−2^, 450 MHz). The temperature change of EuMOF, ZIF, EuMOF@ZIF were monitored by infrared thermal imager (Additional file 1: Figures S8A, S9A, Fig. 2A). A minute-by-minute snapshot of EuMOF@ZIF nanocomposite was shown below. The temperature rising curves of different concentrations of the EuMOF@ZIF nanocomposite were shown in the Fig. 2B. The comparison of temperature rising of different concentrations EuMOF, ZIF and EuMOF@ZIF nanocomposite was shown in the Fig. 2C. The results showed that the temperature change of EuMOF@ZIF nanocomposite at 2, 4, 8 mg/mL was 22.7, 28.1, 35.8 ℃, respectively (Additional file 1: Figure S8D). The increased temperature of EuMOF@ZIF nanocomposite at 2, 4, 8 mg/mL was 1.9, 7.3, 15 ℃ higher than that of the control group, respectively. The increased temperature of EuMOF at 2, 4, 8 mg/mL was 4.8, 4.9, 9.5 ℃ higher than that of the control group, respectively. The increased temperature of ZIF at 2, 4, 8 mg/mL was 1.9, 7.6, 14.6 ℃ higher than that of the control group, respectively. The result showed the temperature rising of EuMOF@ZIF nanocomposite at 8 mg/mL significantly higher than that of EuMOF. We speculate that this is because EZ nanocomposites coated with EuMOF are more stable and difficult to agglomerate at higher concentrations. Similarly, the temperature rising curve of EuMOF and ZIF, the real-time IR thermal image per minute, and the temperature rise comparison histogram were also listed (Additional file 1: Figures S8B, C, S9B, C). The microwave heating effect test showed that EuMOF@ZIF nanocomposite had excellent microwave heating performance.

### Luminescent property evaluation

It has been reported that the characteristic emission wavelength of the lanthanide metal europium ion is around 618 nm, and the emission peak is sharp. First, the emission spectra of nanomaterials at different excitation wavelengths (Ex = 280, 300, 320, 340, 360, 380, 390, 400, 420, 440 nm) were measured. The emission range is 500–750 nm, the excitation end band-pass is 5 nm, the emission end band-pass is 5 nm, and the integration time is 0.05 s. The emission spectrum of each sample showed that the excitation wavelength increased from 280 to 440 nm (Fig. 2D, E). Whether EuMOF or EuMOF@ZIF, the intensity of the maximum emission peak at 618 nm gradually decreased. The relationship between the maximum emission peak intensity and excitation wavelength of EuMOF and EuMOF@ZIF was shown respectively (Additional file 1: Figures S10 and S11). Meanwhile, fluorescence tests showed that ZIF coating did not affect the fluorescence properties of EuMOF. Then, when the excitation wavelength was 280 nm, the solvent was 5% glucose solution, and the fluorescence intensity of the samples (EuMOF, EuMOF@ZIF) with different concentrations was tested, as shown in Fig. 2G, H. Therefore, it is anticipated that EuMOF@ZIF nanocomposite could be a nano-fluorescent probe for tumor diagnosis.

### Drug loading and release

Apatinib is a star targeted drug approved by the US Food and Drug Administration and currently used in first-line chemotherapy. It is loaded into EuMOF@ZIF nanocomposite and enables chemotherapy and microwave ablation to play a synergistic role in the treatment of tumors. The drug loading rate was calculated by testing the characteristic UV–vis absorption of Apatinib. The UV–vis absorption spectra was the characteristic UV–vis absorption of Apatinib in the supernatant of EuMOF@ZIF/AP (Additional file 1: Figure S12). According to the calculation formula in the previous report of our research group[48], the drug loading of EuMOF@ZIF was calculated to be 20.37%, while the drug loading of EuMOF was calculated to be 9.2%. It also demonstrated that the ZIF effectively improved drug-loading rate of the EuMOF@ZIF nanocomposite. In order to understand the drug release of EuMOF@ZIF/AP, drug release experiments were carried out. UV–vis absorption curves of Apatinib drug release of different concentrations were obtained, as shown in Additional file 1: Figure S13, and the standard curve of drug release (Additional file 1: Figure S14) was drawn by absorption peaks of Apatinib drug release of different concentrations. According to the drug release curve, the drug release rate of the EuMOF@ZIF/AP nanocomposite was calculated to be 55.68% under neutral physiological conditions (Fig. 2F). The drug release of EuMOF@ZIF in PBS solutions (pH = 6.5) was investigated and the data was shown in Additional file 1: Figure S14. The drug release rate of the EuMOF@ZIF/AP nanocomposite was calculated to be 75.10% under pH = 6.5, the result showed that EuMOF@ZIF/AP nanocomposite had a faster drug release under weakly acidic conditions than under neutral conditions (Additional file 1: Figure S15). In addition, the results of microwave stimulated drug release experiments showed that the drug release rate reached 84.40% (Fig. 2I). The experimental results showed that EuMOF@ZIF nanocomposite could achieve loading and release of the drug, and the ZIF effectively improved drug loading rate of the EuMOF@ZIF nanocomposite.

Moreover, Apatinib is known to down-regulate vascular epidermal growth factor (VEGF) expression in tumor vessels. VEGF immunofluorescence was used to detect whether EZAP could down-regulate the expression of VEGF or not. Strong green fluorescence was observed in the control group, and obvious fluorescence signal was also observed in the EZP group, while the green fluorescence signal was almost not obvious in the Apatinib group and EZAP group (Additional file 1: Figure S16). The results showed that EZAP down-regulated VEGF expression, but EZP did not, further indicating the successful loading of Apatinib.

### In vitro microwave thermo-chemotherapy effect

**Fig. 3:**
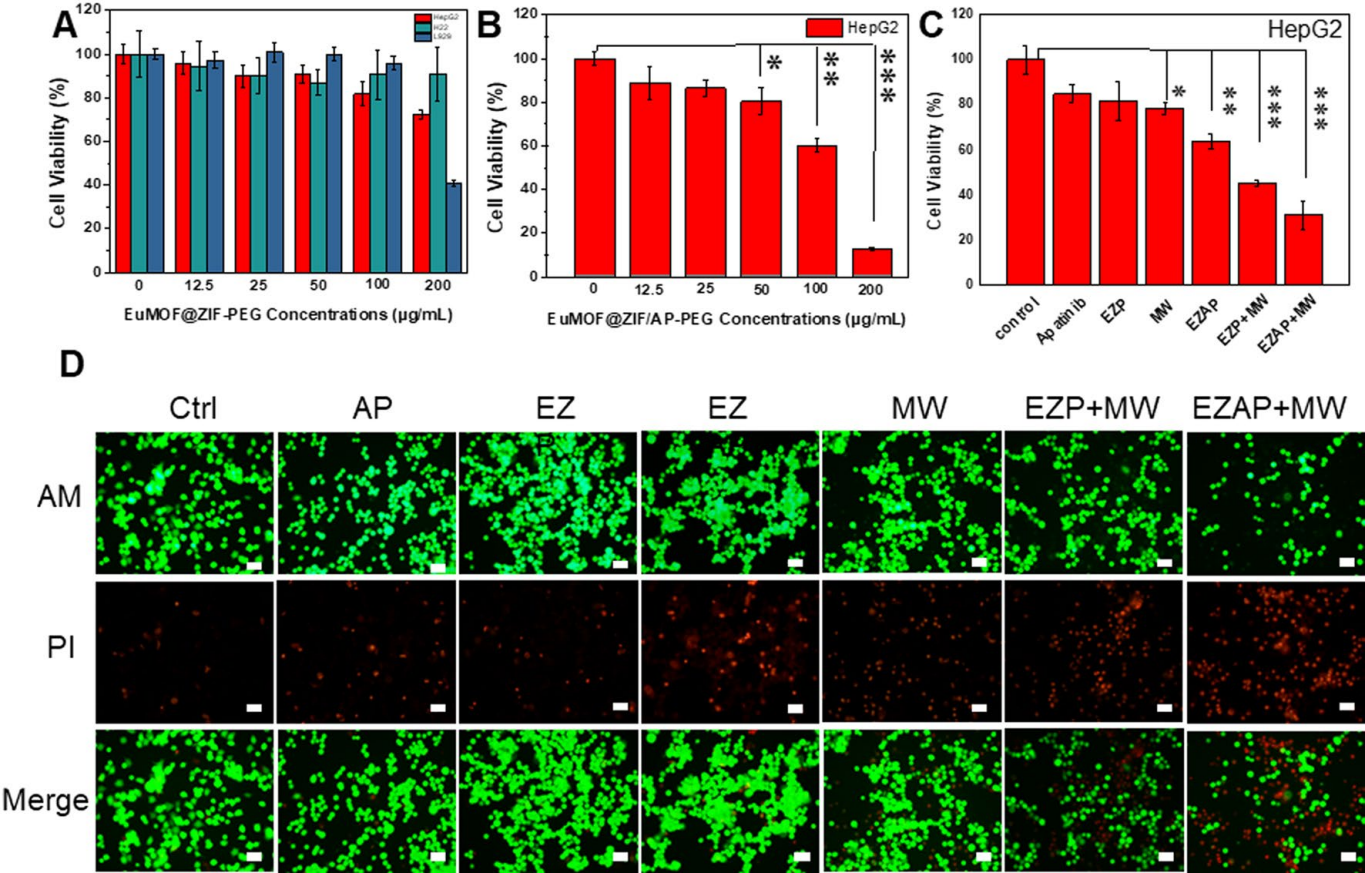
Cytotoxicity and in vitro microwave thermo-chemotherapy effect. **A** relative cell viability of EuMOF@ZIF after PEG modification to cancer cells and normal cells, respectively. **B** Inhibition of different concentrations of EuMOF@ZIF after drug loading on cancer cells. **C** Relative cell viability, **D** Living-dead staining of cancer cells under different treatments (control, free Apatinib, EZP, MW, EZAP, EZP + MW, EZAP + MW). The scale bar is 50 μm. ***p < 0.001, **p < 0.01, *p < 0.05

In order to better investigate the subsequent biological applications, the PEG modification of the nanocomposite was used to improve the biocompatibility. First, the size distribution of EuMOF@ZIF-PEG (EZP) nanocomposite was tested as ~ 349 nm, while the zeta-potential of EuMOF@ZIF-PEG nanocomposite is + 14.2 mV, which implied the size of EuMOF@ZIF nanocomposites modified by PEG was negligible, but its potential decreased, indicating that the modification was successful. (Additional file 1: Figures S17, S18). Next, Hepg2 and H22 were used as tumor cell models, and L929 as normal cell models. MTT test results showed that the EuMOF@ZIF-PEG nanocomposite had good biocompatibility for both tumor and normal cells (Fig. 3A). Additionally, the relative cell viability of EuMOF was tested and the results showed good biosafety (Additional file 1: Figures S19–S21). As a drug carrier, it is endowed with the chemotherapeutic effect by the EuMOF@ZIF/AP-PEG nanocomposite obtained after loading Apatinib. Firstly, the relative cell viability of the EuMOF@ZIF/AP-PEG nanocomposite on HepG2 tumor cells at different concentrations were investigated to evaluate the efficacy of chemotherapy. The experimental results were shown in Fig. 3B. The relative cell viability was above 80% at 0–50 μg/mL, which could not achieve the therapeutic effect. Compared with the EuMOF@ZIF-PEG nanocomposite, the relative cell viability decreased to 60.04% at 100 μg/mL and 12.53% at 200 μg/mL. These results indicate that EuMOF@ZIF/AP-PEG nanocomposites with a concentration of 100 μg/mL have definitely killing effect on tumor cells (Fig. 3B). Therefore, the therapeutic dose chosen in this paper was 100 μg/mL. The results showed that Apatinib, as a chemotherapy drug, had a certain inhibitory effect on HepG2 tumor cells.

Next, we performed tumor cell inhibition experiments in vitro. Compared with the control group, the relative cell viability of free Apatinib group, EuMOF@ZIF-PEG group, MW group, EuMOF@ZIF/AP-PEG group, EuMOF@ZIF-PEG + MW group and EuMOF@ZIF/AP-PEG + MW group were 84.9%, 81.8% and 78.5, 63.8%, 45.1%, 31.0%, respectively (Fig. 3C). The results showed that EuMOF@ZIF/AP-PEG + MW group had the best tumor inhibition effect. Under microwave irradiation, it showed the combination of microwave sensitization of EuMOF@ZIF/AP-PEG and chemotherapy. In addition, H22 tumor cell inhibition experiments were conducted, as shown in Additional file 1: Figures S22 and S23. Compared with the control group, the relative cell viability of EuMOF@ZIF-PEG group, EuMOF@ZIF/AP-PEG group, MW group, EuMOF@ZIF-PEG + MW group, and EuMOF@ZIF/AP-PEG + MW group were 88.7%, 88.5%, 66.1%, 36.3%, 12.3%, respectively. The experimental results showed that similar results were obtained compared with the HepG2 experiment.

To further verify the inhibitory effect of nanocomposite on HepG2 cells and H22 cells by the Calcein-AM/PI staining. The experimental results of HepG2 cells were shown in Fig. 3D. First, the red fluorescence in the control group is the least, indicating the least number of dead cells. Secondly, the red fluorescence intensity of free Apatinib group and EuMOF@ZIF-PEG group was almost the same, but stronger than that of the control group. In addition, the fluorescence intensity of the MW group and the EuMOF@ZIF/AP-PEG group were similar, but the fluorescence intensity of the MW group and the EuMOF@ZIF/AP-PEG group was significantly increased compared with the previous two groups. Finally, compared with the other groups, bright and intense red fluorescence could be seen in EuMOF@ZIF/AP-PEG + MW group, followed by the EuMOF@ZIF-PEG + MW group, indicating the highest eliminating ability to tumors in the EuMOF@ZIF/AP-PEG + MW group. In the Calcein-AM staining of living cells, compared with the control group, the green fluorescence intensity of free Apatinib group, EuMOF@ZIF-PEG group, MW group, EuMOF@ZIF/AP-PEG group, EuMOF@ZIF-PEG + MW group, and EuMOF@ZIF/AP-PEG + MW group were in order. The fluorescence intensity of EuMOF@ZIF/AP-PEG + MW group decreased most obviously, indicating that the number of surviving cells was the least. Similar data were obtained from the staining experiment for HepG2 cells and the staining experiment for suspension H22 cells (Additional file 1: Figure S24). In general, the results of Calcein-AM/PI live staining showed that EuMOF@ZIF/AP-PEG + MW group had the strongest tumor inhibition effect, which reflected the microwave sensitization of the EuMOF@ZIF/AP-PEG nanocomposite and the combined effect of chemotherapy under microwave irradiation.

### In vivo liver cancer microwave thermo-chemotherapy

**Fig. 4:**
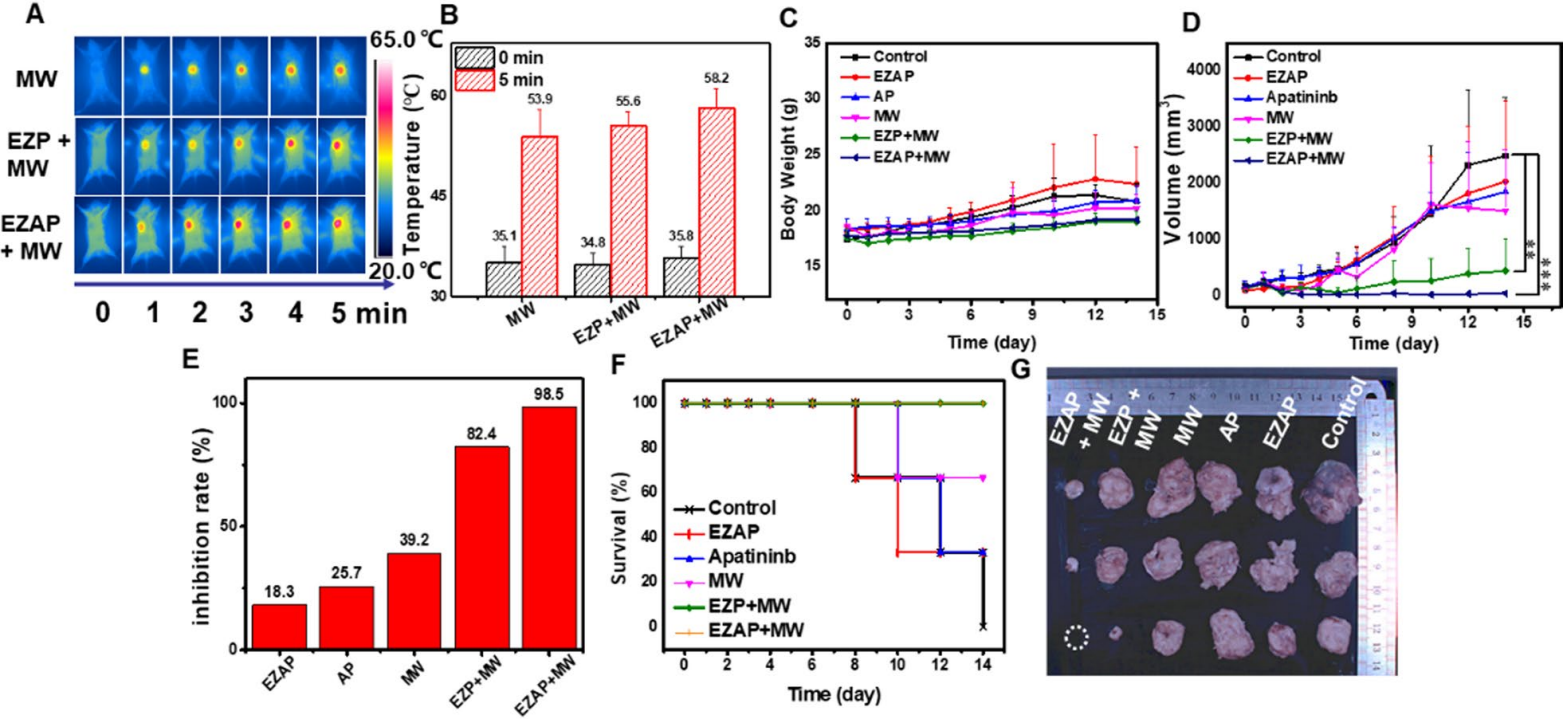
Microwave thermo-chemotherapy. **A** Infrared thermal images of tumor-bearing mice under different treatment methods (MW, EZP + MW, EZAP + MW) under microwave irradiation for 5 min. **B** Temperature of tumor-bearing mice with different treatment methods (MW, EZP + MW, EZAP + MW) at the beginning and end of microwave irradiation. **C** The body weight changes, **D** Tumor volume changes of different groups of tumor-bearing mice within 15 days. ***p < 0.001, **p < 0.01, *p < 0.05. **E** Inhibition rate in each group after 15 days of treatment compared to the control group. **F** Survival rates of different groups of tumor-bearing mice within 15 days. **G** In vitro tumor images of different groups of tumor-bearing mice on the last day are control group, EZAP group, Apatinib group, MW group, EZP + MW group, and EZAP + MW group from top to bottom, white dots indicate that the mouse tumor has been cured without recurrence

The above results showed that EuMOF@ZIF/AP-PEG nanocomposite was designed and characterized, which possessed high microwave sensitization, fluorescence imaging function and good drug loading in vitro. In order to verify the above properties of the material in vivo, H22 tumor-bearing mice were used as animal experimental model to evaluate the tumor inhibition effect. All animal experiments were carried out in accordance with the agreement of ethics committee of Harbin Medical University Cancer Hospital. Next, the experimental design was divided into 6 groups: control group (without any treatment), EuMOF@ZIF/AP-PEG group (containing drug material), free Apatinib group, MW group, EuMOF@ZIF-PEG + MW group (without drug material and microwave), and EuMOF@ZIF/AP-PEG + MW group (including drug material and microwave). The changes of body weight showed that the body weight of mice in each group did not fluctuate much within 15 days (Fig. 4C), demonstrating the biocompatibility and biosafety of the nanocomposite in vivo. During microwave irradiation, the tumor temperature changes in the MW group, EuMOF@ZIF-PEG + MW group and EuMOF@ZIF/AP-PEG + MW group were monitored in real time. With the increase of microwave irradiation time, the tumor temperature in each group increased, and it can be seen that the temperature rise of the experimental group was obviously higher than that of the control group. It is further proved that the EuMOF@ZIF/AP-PEG nanocomposite has good microwave sensitization performance under microwave irradiation (Fig. 4A, B). The tumor volume of mice in the EuMOF@ZIF/AP-PEG + MW group significantly less than the control group after 14 days (Fig. 4D). Moreover, the inhibitory rate of EuMOF@ZIF/AP-PEG + MW group was 98.5% compared with the control group according to the formula of inhibitory rate (Fig. 4E), and the results of tumor inhibition experiment in vivo showed that the EuMOF@ZIF /AP-PEG + MW group had an excellent tumor inhibition effect. Images of different groups of tumor-bearing mice at 0 and 14 days, and tumor tissue sections in vitro (Additional file 1: Figure S28 and Fig. 4H) showed that the MW group, the drug-containing material without microwave and the simple material combined with microwave group are higher than the control group, but the therapeutic effect is lower than the experimental group, indicating that microwave-thermal therapy combined with chemotherapy could effectively inhibit tumor growth and enhance the effect of microwave treatment. In addition, as shown in Fig. 4F, G, the survival rate of both EZAP + MW group and EZP + MW group was 100%, the survival rate of the MW group was 66.6%, the survival rate of the EZAP group and the AP group was 33.3%, and the survival rate of the control group was 0%. In a word, in vivo tumor inhibition experiment results showed that thermal therapy combined with chemotherapy under microwave irradiation could achieve a good effect on tumor treatment.

**Fig. 5:**
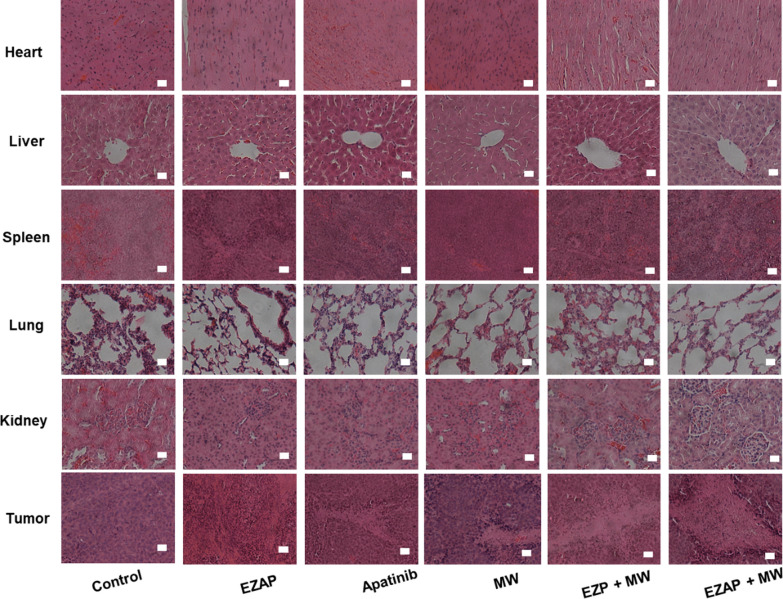
HE staining section of the main organs and tumors of tumor-bearing mice. The scale bar is 50 μm

In order to further observe and analyze the biological safety of the nanocomposite in mice, Hematoxylin eosin staining was performed (Fig. 5). The results showed that there was no obvious organ damage in the tissue sections of heart, liver, spleen, lung and kidney tumors in each group. However, in tumor tissue, the EuMOF@ZIF/AP-PEG + MW group had significant large area of cell damage, followed by the EuMOF@ZIF-PEG + MWgroup, EuMOF@ZIF/AP-PEG group and MW group had a small amount of cell damage, and the control group had no cell damage. In vivo acute toxicity tests were used to evaluate the biosafety of EZAP nanocomposite. Body weight change analysis of EZAP group showed no significant abnormalities in routine blood test and blood biochemical analysis (Additional file 1: Figures S25-27). This suggests that EZAP has good biocompatibility and could be further used in vivo therapy.

### In vivo fluorescence imaging

**Fig. 6:**
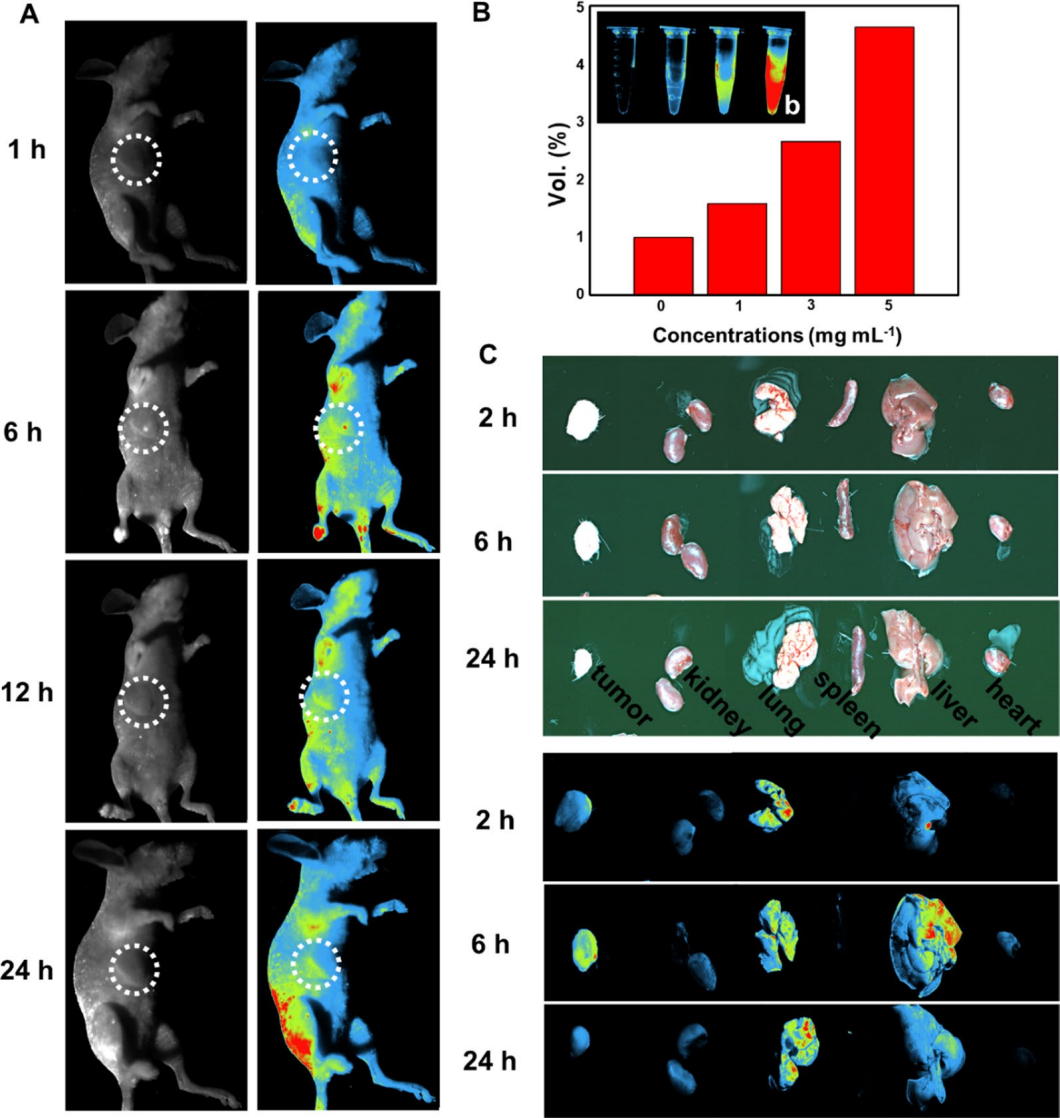
In vivo Fluorescence Imaging. Fluorescence signal images of **A** tumor sites **C** the main organs and ex vitro tumor in tumor-bearing nude mice at different time. **B** Fluorescence signal quantitative diagram, **b** fluorescence signal diagram of EZAP with different concentrations excited at 365 nm

Encouraged by the fluorescence properties of the EuMOF@ZIF nanocomposite mentioned above, we performed fluorescence imaging monitoring of mouse tumors in vivo. The emission spectra of the EuMOF@ZIF-PEG nanocomposite at different excitation wavelengths were studied. The emission map of the EuMOF@ZIF-PEG nanocomposite showed that the intensity of the maximum emission peak at 618 nm gradually decreased as the excitation wavelength gradually increased. In addition, the relationship between the maximum emission peak value and the excitation wavelength was plotted (Additional file 1: Figures S29, S30), the fluorescence test showed that PEG modification did not affect the fluorescence properties of the EuMOF@ZIF nanocomposite. Furthermore, fluorescence signal images of different material concentrations were obtained in in vivo imaging experiments (Fig. 6A). As well as the fluorescence signal images collected at different time points after the injection of EuMOF@ZIF/AP-PEG nanocomposite into the tail vein of mice (Fig. 6B). The signal of the EuMOF@ZIF/AP-PEG nanocomposite in mouse tumor was enhanced with the extension of time after the injection of caudal vein in a certain period of time. At 6 h, there were obvious fluorescence signals in the tumor sites of mice. Then, over time, the fluorescence signal of EuMOF@ZIF/AP-PEG nanocomplexes at the tumor site in mice gradually decreased. To understand the effect of nanocomposite on the organs of mice, we dissected the main organs and tumors of mice at different time points to observe the change of fluorescence signal intensity. The fluorescence signal at the tumor site was the strongest after 6 h material injection (Fig. 6C), which was consistent with the experimental results in vivo. In addition, there were also obvious fluorescence signals in the liver and lung of mice, followed by the kidney and heart, and the spleen. In general, the EuMOF@ZIF/AP-PEG nanocomposite as fluorescent probes had demonstrated their application prospects in vivo imaging and demonstrated their diagnostic potential.

## Conclusions

In summary, a novel theranostic nanoplatform (EuMOF@ZIF/AP-PEG nanocomposite) was designed and characterized, which had high microwave sensitization, fluorescence imaging function and good drug loading. MTT assay showed that the concentration of EZAP nanocomposite was 100 μg/mL and had low biotoxicity. In vitro tumor cell inhibition experiments showed that thermal therapy combined with chemotherapy under microwave irradiation had the strongest inhibitory effect on tumor cell growth. Compared with 100% in the control group, the relative activity of cells was reduced to 31%. Through the treatment experiment of H22 tumor-bearing mice, it was further verified that the EZAP nanocomposite had good microwave sensitization. The EZAP nanocomposite assisted MW irradiation, thermal therapy combined with chemotherapy could achieve a good effect on tumor treatment, showing excellent anti-tumor effect, tumor inhibition rate of 98.5%, indicating that the EZAP nanocomposite had enormous potential for the combined treatment of microwave-thermal therapy and chemotherapy. The fluorescence imaging in vivo experiment showed that the EZAP nanocomposite could be used as a potential fluorescent probe. The EZAP nanocomposite showed was strongest fluorescence signal after 6 h of caudal vein injection, which could monitor the treatment of tumor and realize the integrated function of diagnosis and treatment. Our design strategy advances the lanthanide MOF in vivo imaging research. It also provides a novel procedure for the biological application of lanthanide MOF.

Taken together, the EZAP nanocomposite demonstrated the potential of microwave thermo-chemotherapy therapy and in vivo imaging, and provided further inspiration for future imaging monitoring therapies.

## Supplementary Information

**Additional file 1: Figure S1.** SEM image and TEM image of EuMOF. **Figure S2.** The size distribution of EuMOF. **Figure S3.** The zeta-potential of EuMOF. **Figure S4.** The size distribution of EuMOF-PVP. **Figure S5.** The zeta-potential of EuMOF-PVP. **Figure S6.** The stability of EuMOF@ZIF and EuMOF at different time points in PBS (pH = 7.4), the scale bar was 1000 nm. **Figure S7.**The photos of EuMOF and EZ in different solution (1, PBS solution at pH 5.7, 2, PBS solution at pH 7.4) at different time after stand still. **Figure S8.** Infrared thermal images, temperature variation curves, temperature rise histogram of EuMOF (0, 2, 4, 8 mg/mL) under microwave irradiation for 5 min. Temperature rise histogram of EuMOF@ZIF (0, 2, 4, 8 mg/mL) under microwave irradiation for 5 min. **Figure S9.** (A) Infrared thermal images, (B) temperature variation curves, (C) temperature rise histogram of ZIF (0, 2, 4, 8 mg/mL) under microwave irradiation for 5 min. **Figure S10.** The diagram between the maximum emission peak intensity and the excitation wavelength of EuMOF. **Figure S11.** The diagram between the maximum emission peak intensity and the excitation wavelength of EuMOF@ZIF. **Figure S12.** Ultraviolet absorption curve of supernatant obtained by centrifugal washing after 1, EuMOF@ZIF loading drugs, 2, EuMOF loading drugs. **Figure S13.** Ultraviolet absorption curves of Apatinib at different concentrations. **Figure S14.** Standard curve of Apatinib at different concentrations in drug release solvent. **Figure S15.** Drug release rate of EZ loaded apatinib under pH = 6.5. **Figure S16.** Immunofluorescence images of VEGF antibody expression after co-incubation with HepG2 in different groups. **Figure S17.** The size distribution of EuMOF@ZIF-PEG. **Figure S18.** The zeta-potential of EuMOF@ZIF-PEG. **Figure S19.** The relative cell viability of EuMOF to HepG2 tumor cells. **Figure S20.** The relative cell viability of EuMOF to L929 cells. **Figure S21.** The relative cell viability of EuMOF to H22 tumor cells. **Figure S22.** Inhibition of different concentrations of EuMOF@ZIF after drug loading on H22 cells. **Figure S23.** Relative cell viability of H22 tumor cells under different treatments (control, EZP, MW, EZAP, EZP + MW, EZAP + MW). **Figure S24.** Living-dead dyeing of H22 tumor cells under different treatments (control, EZP, MW, EZAP, EZP + MW, EZAP + MW). **Figure S25.** Weight change of mice in acute toxicity test after injecting different doses (0, 50, 75 mg/kg) of EZAP nanocomposites. **Figure S26.** Blood biochemical analysis result of EZAP nanocomposites. **Figure S27.** Blood routine test result of EZAP nanocomposites (0, 25, 50 mg/kg). **Figure S28.** Representative images of different groups of tumor-bearing mice at 0 and 14 days respectively. **Figure S29.** Emission spectra of EuMOF@ZIF-PEG at different excitation wavelengths. **Figure S30.** The diagram between the maximum emission peak intensity and the excitation wavelength of EuMOF@ZIF-PEG.

## Data Availability

The datasets and materials used in the study are available from the corresponding author.
