# Supplementary material for: Lanthanide europium MOF nanocomposite as the theranostic nanoplatform for microwave thermo-chemotherapy and fluorescence imaging
Source: J Nanobiotechnology. 2022 Mar 15;20:133. doi: 10.1186/s12951-022-01335-7 (PMC8922785; doi:10.1186/s12951-022-01335-7)
Supplement: Supplementary file 1 — Additional file 1: Figure S1. SEM image and TEM image of EuMOF. Figure S2. The size distribution of EuMOF. Figure S3. The zeta-potential of EuMOF. Figure S4. The size distribution of EuMOF-PVP. Figure S5. The zeta-potential of EuMOF-PVP. Figure S6. The stability of EuMOF@ZIF and EuMOF at different time points in PBS (pH = 7.4), the scale bar was 1000 nm. Figure S7.The photos of EuMOF and EZ in different solution (1, PBS solution at pH 5.7, 2, PBS solution at pH 7.4) at different time after stand still. Figure S8. Infrared thermal images, temperature variation curves, temperature rise histogram of EuMOF (0, 2, 4, 8 mg/mL) under microwave irradiation for 5 min. Temperature rise histogram of EuMOF@ZIF (0, 2, 4, 8 mg/mL) under microwave irradiation for 5 min. Figure S9. (A) Infrared thermal images, (B) temperature variation curves, (C) temperature rise histogram of ZIF (0, 2, 4, 8 mg/mL) under microwave irradiation for 5 min. Figure S10. The diagram between the maximum emission peak intensity and the excitation wavelength of EuMOF. Figure S11. The diagram between the maximum emission peak intensity and the excitation wavelength of EuMOF@ZIF. Figure S12. Ultraviolet absorption curve of supernatant obtained by centrifugal washing after 1, EuMOF@ZIF loading drugs, 2, EuMOF loading drugs. Figure S13. Ultraviolet absorption curves of Apatinib at different concentrations. Figure S14. Standard curve of Apatinib at different concentrations in drug release solvent. Figure S15. Drug release rate of EZ loaded apatinib under pH = 6.5. Figure S16. Immunofluorescence images of VEGF antibody expression after co-incubation with HepG2 in different groups. Figure S17. The size distribution of EuMOF@ZIF-PEG. Figure S18. The zeta-potential of EuMOF@ZIF-PEG. Figure S19. The relative cell viability of EuMOF to HepG2 tumor cells. Figure S20. The relative cell viability of EuMOF to L929 cells. Figure S21. The relative cell viability of EuMOF to H22 tumor cells. Figure S22. Inhibition of differ [file 12951_2022_1335_MOESM1_ESM.docx]

**Supporting information**

**Lanthanide europium MOF nanocomposite as the theranostic nanoplatform for microwave thermo-chemotherapy and fluorescence imaging**

Lirong Zhao^1,4^, Wei Zhang^3^, Qiong Wu^1^, Changhui Fu^1^, Xiangling Ren^1^, Kongpeng Lv ^3^, Tengchuang Ma^2,*^, Xudong Chen^3^, Longfei Tan^1,*^, Xianwei Meng^1,*^

^1^Laboratory of Controllable Preparation and Application of Nanomaterials, Technical Institute of Physics and Chemistry, Chinese Academy of Sciences; CAS Key Laboratory of Cryogenics, Technical Institute of Physics and Chemistry, No. 29 East Road Zhongguancun, Beijing 100190, P. R. China

^2^Department of Nuclear Medicine, Harbin Medical University Cancer Hospital, Nangang District, Harbin, Heilongjiang Province 150086, P.R. China

^3^Department of Interventional Radiology, Shenzhen People's Hospital (The Second Clinical Medical College, Jinan University; The First Affiliated Hospital, Southern University of Science and Technology), Shenzhen 518020, Guangdong, China

^4^University of Chinese Academy of Sciences, Beijing 100049, P.R. China

*Correspondence: [mengxw@mail.ipc.ac.cn](mailto:mengxw@mail.ipc.ac.cn); [longfeitan@mail.ipc.ac.c](mailto:longfeitan@mail.ipc.ac.c); matengchuang1988@126.com.

^1^Laboratory of Controllable Preparation and Application of Nanomaterials, CAS Key Laboratory of Cryogenics, Technical Institute of Physics and Chemistry, Chinese Academy of Sciences, No. 29 East Road Zhongguancun, Beijing 100190, P. R. China


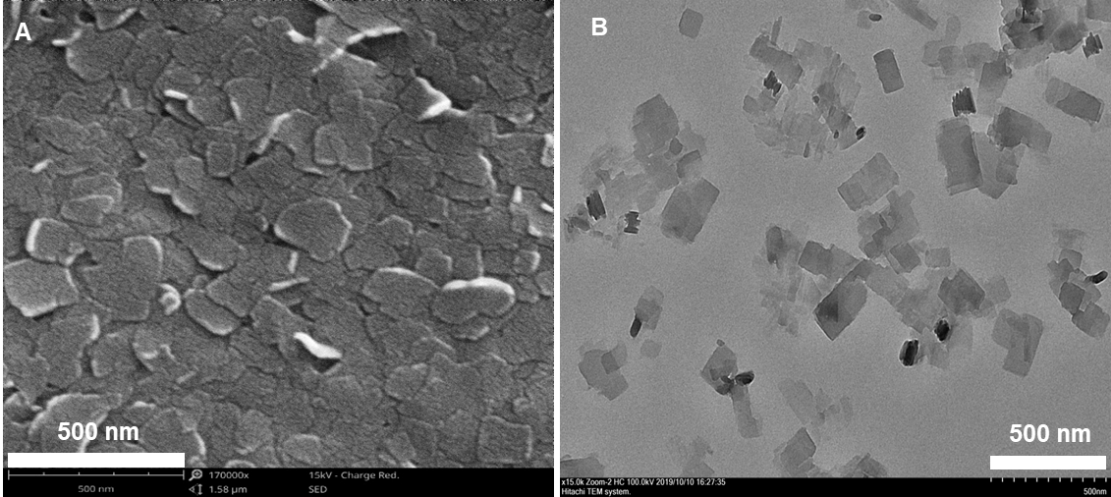


Figure S1. (A) SEM image and (B)TEM image of EuMOF.

Figure S2. The size distribution of EuMOF.

Figure S3. The zeta-potential of of EuMOF.

Figure S4. The size distribution of EuMOF-PVP.

Figure S5. The zeta-potential of of EuMOF-PVP.


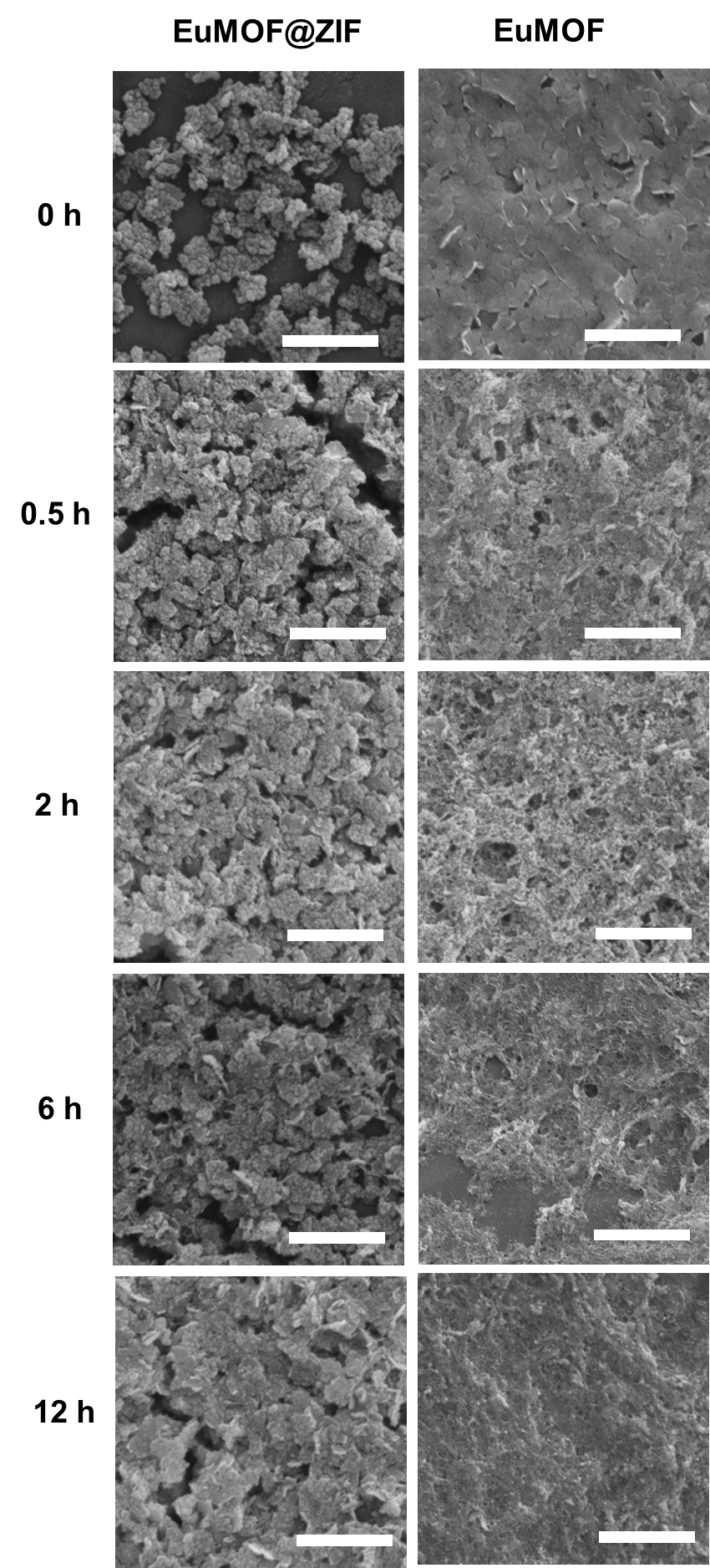


Figure S6. The stability of EuMOF@ZIF and EuMOF at different time points in PBS (pH = 7.4), the scale bar was 1000 nm.


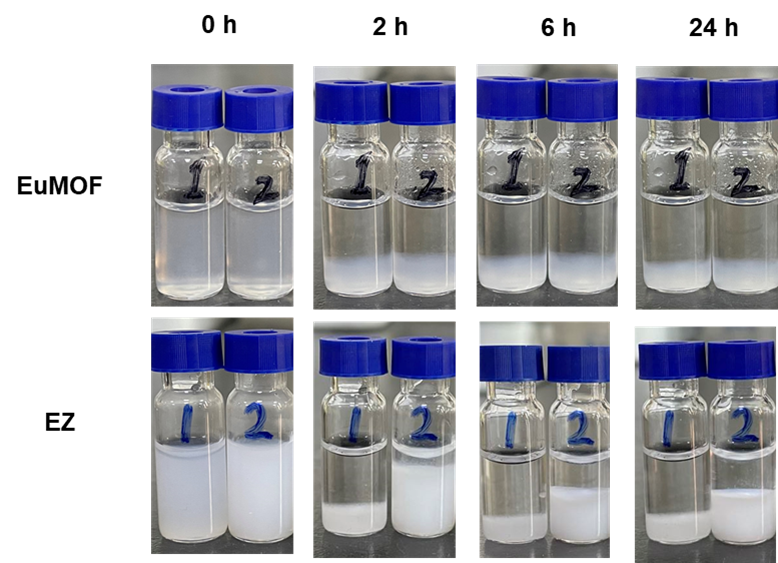


Figure S7. The photos of EuMOF and EZ in different solution (1, PBS solution at pH 5.7, 2, PBS solution at pH 7.4) at different time after stand still.


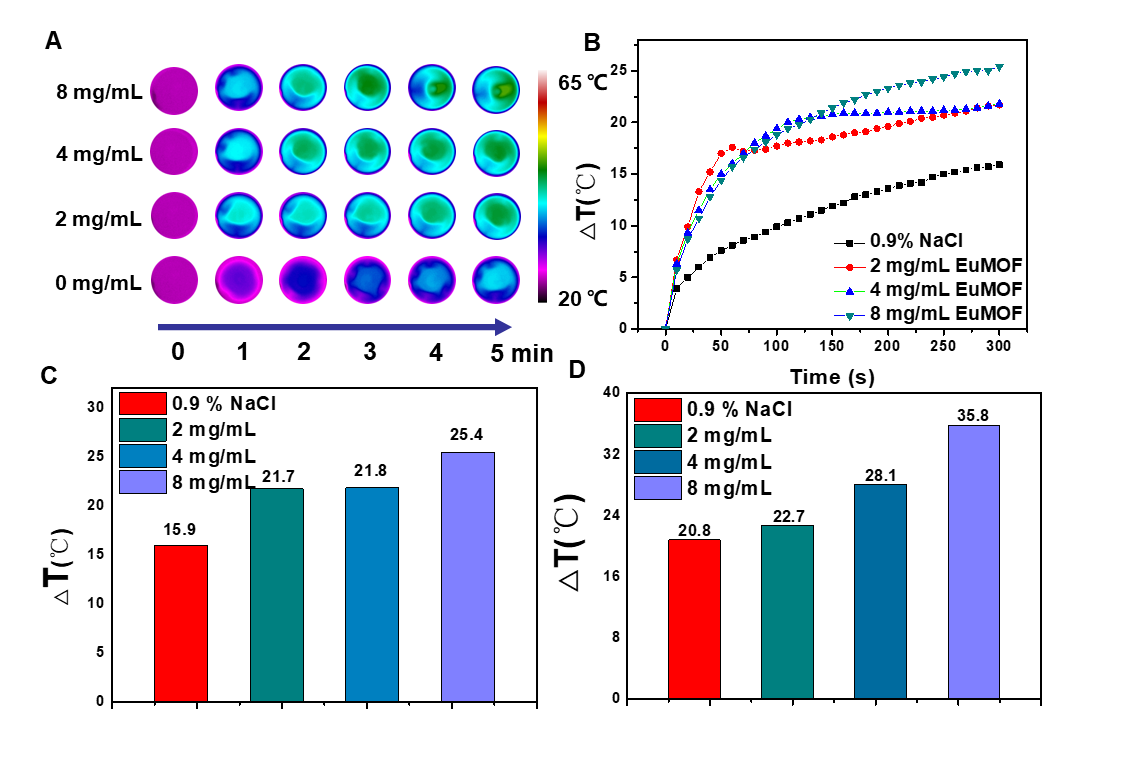


Figure S8. (A) Infrared thermal images, (B) temperature variation curves, (C) temperature rise histogram of EuMOF (0, 2, 4, 8 mg/mL) under microwave irradiation for 5 min. (D) Temperature rise histogram of EuMOF@ZIF (0, 2, 4, 8 mg/mL) under microwave irradiation for 5 min.


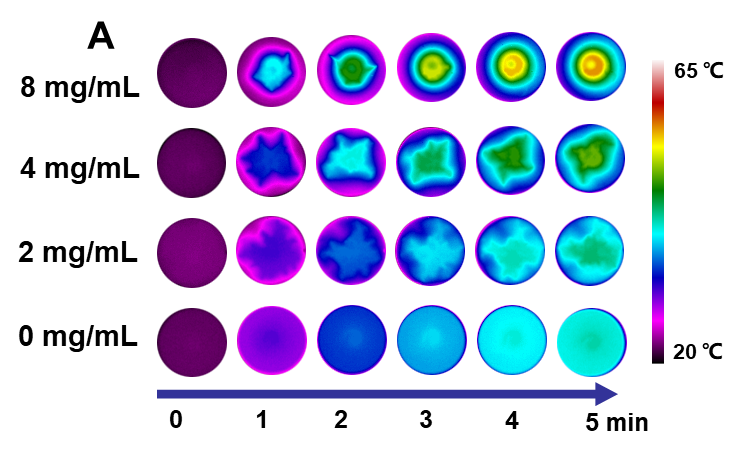


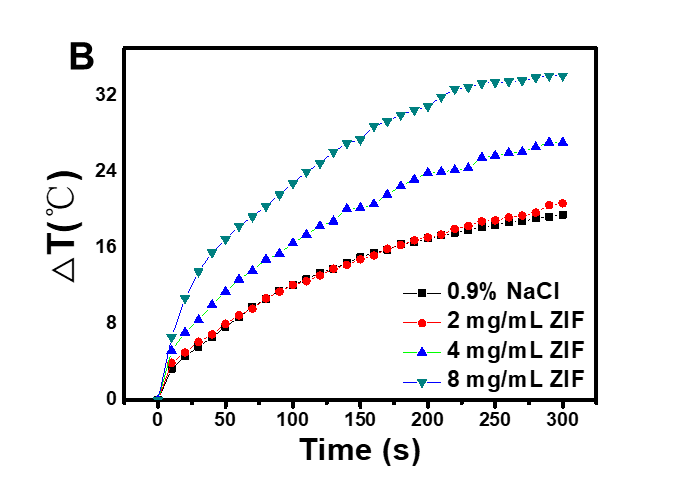


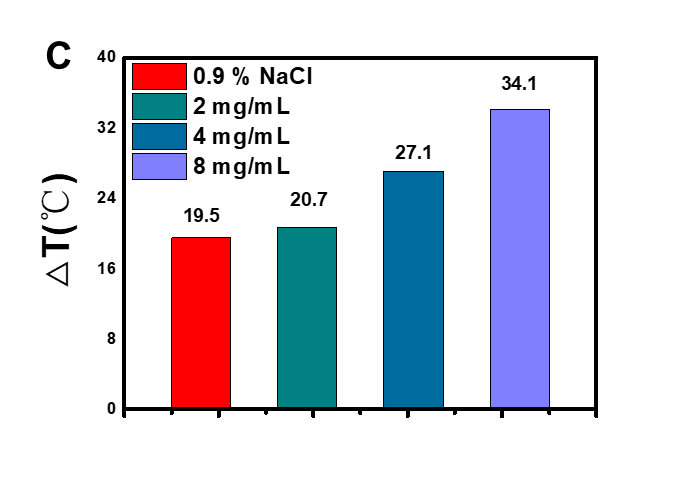


Figure S9. (A) Infrared thermal images, (B) temperature variation curves, (C) temperature rise histogram of ZIF (0, 2, 4, 8 mg/mL) under microwave irradiation for 5 min.

Figure S10. The diagram between the maximum emission peak intensity and the excitation wavelength of EuMOF.

Figure S11. The diagram between the maximum emission peak intensity and the excitation wavelength of EuMOF@ZIF.

Figure S12. Ultraviolet absorption curve of supernatant obtained by centrifugal washing after 1. EuMOF@ZIF loading drugs, 2. EuMOF loading drugs.

Figure S13. Ultraviolet absorption curves of apatinib at different concentrations.

Figure S14. Standard curve of apatinib at different concentrations in drug release solvent.

Figure S15. Drug release rate of EZ loaded apatinib under pH=6.5.


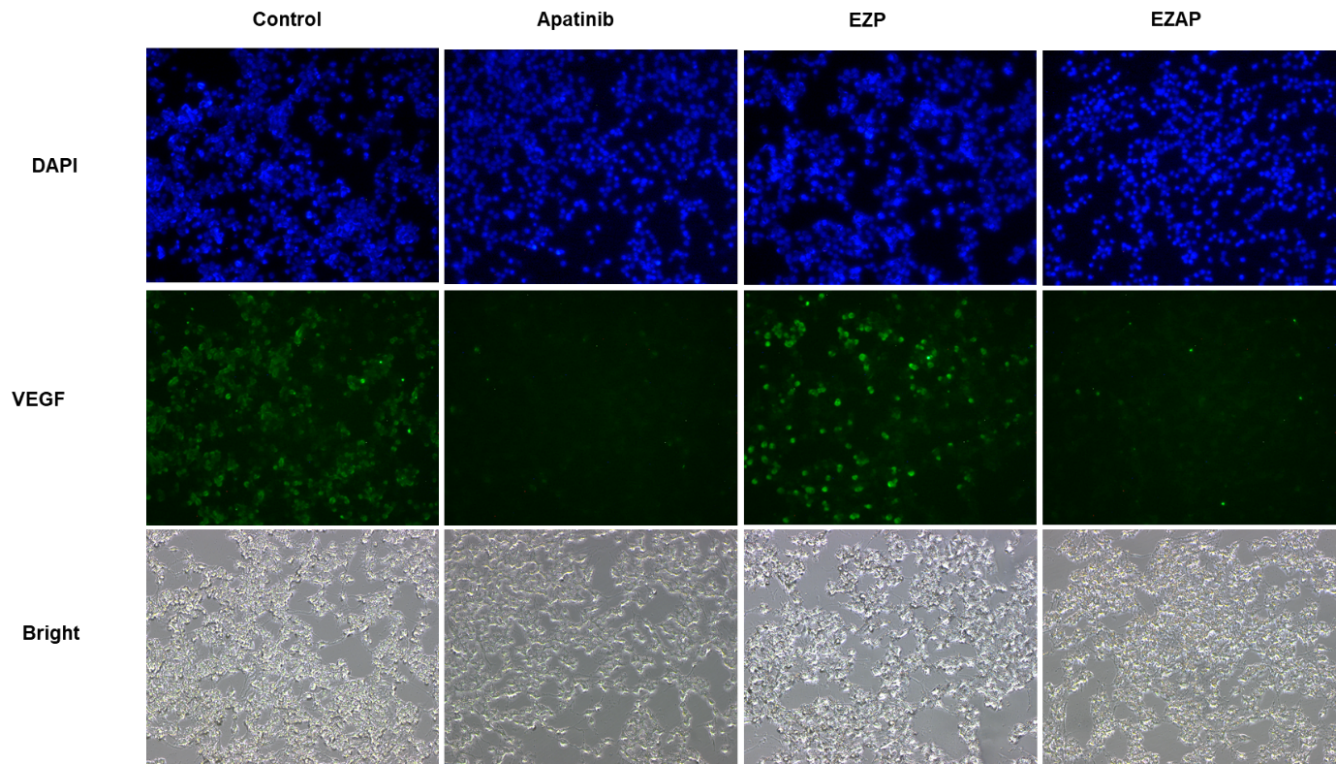


Figure S16. Immunofluorescence images of VEGF antibody expression after co-incubation with HepG2 in different groups.

Figure S17. The size distribution of EuMOF@ZIF-PEG.

Figure S18. The zeta-potential of EuMOF@ZIF-PEG.

Figure S19. The relative cell viability of EuMOF to HepG2 tumor cells.

Figure S20. The relative cell viability of EuMOF to L929 cells.

Figure S21. The relative cell viability of EuMOF to H22 tumor cells.

Figure S22. Inhibition of different concentrations of EuMOF@ZIF after drug loading on H22 cells.

Figure S23. Relative cell viability of H22 tumor cells under different treatments (control, EZP, MW, EZAP, EZP + MW, EZAP + MW).


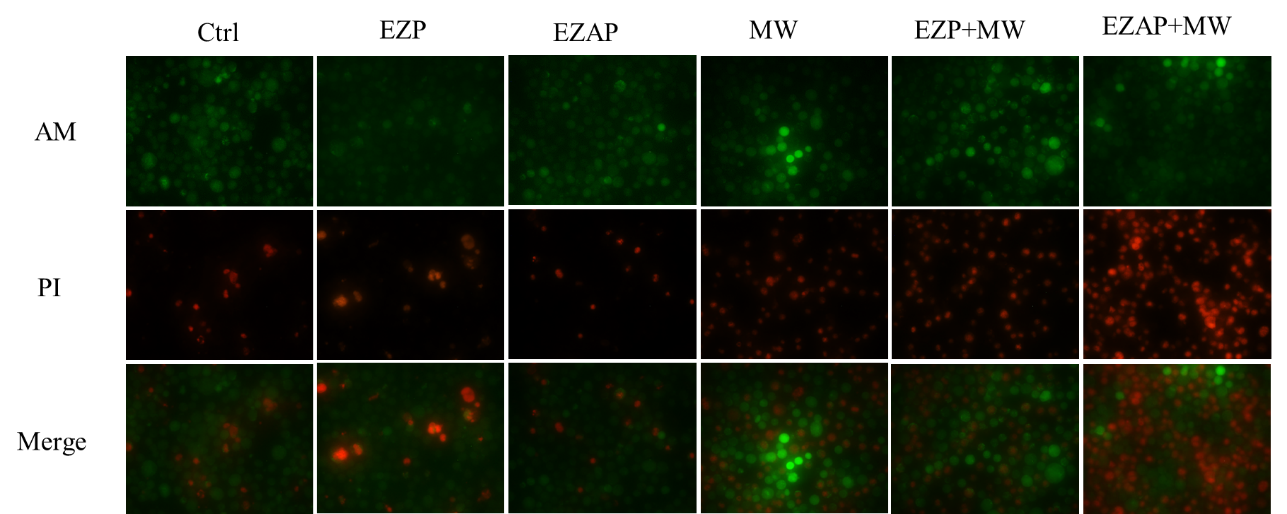


Figure S24. Living-dead dyeing of H22 tumor cells under different treatments (control, EZP, MW, EZAP, EZP + MW, EZAP + MW).

Figure S25. Weight change of mice in acute toxicity test after injecting different doses (0, 50, 75 mg/kg) of EZAP nanocomposites.


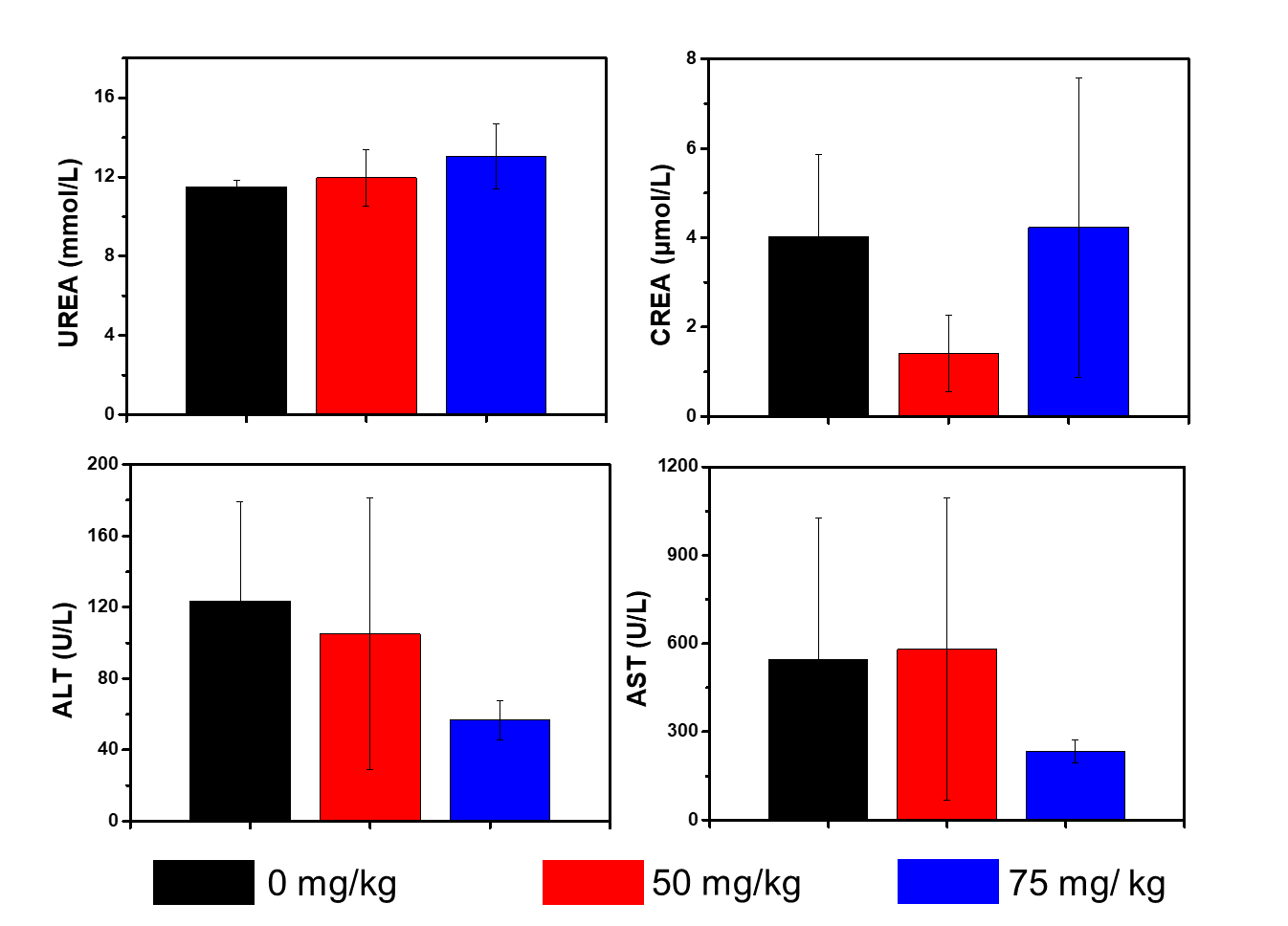


Figure S26. Blood biochemical analysis result of EZAP nanocomposites (including UREA, CREA, ALT and AST).


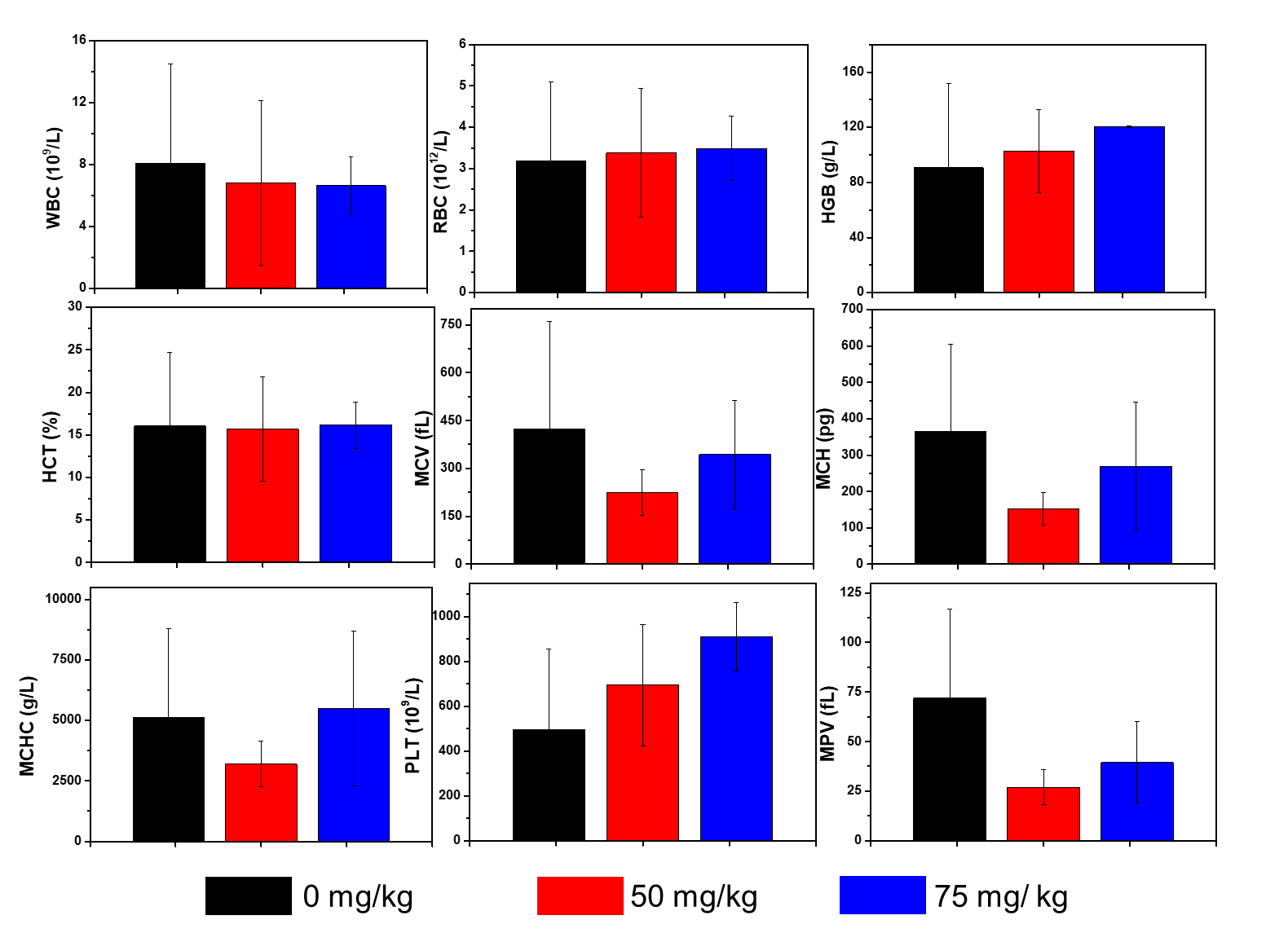


Figure S27. Blood routine test result of EZAP nanocomposites (0, 25, 50 mg/kg) (including WBC, RBC, HGB, HCT, MCV, MCH, MCHC, PLT, and MPV).


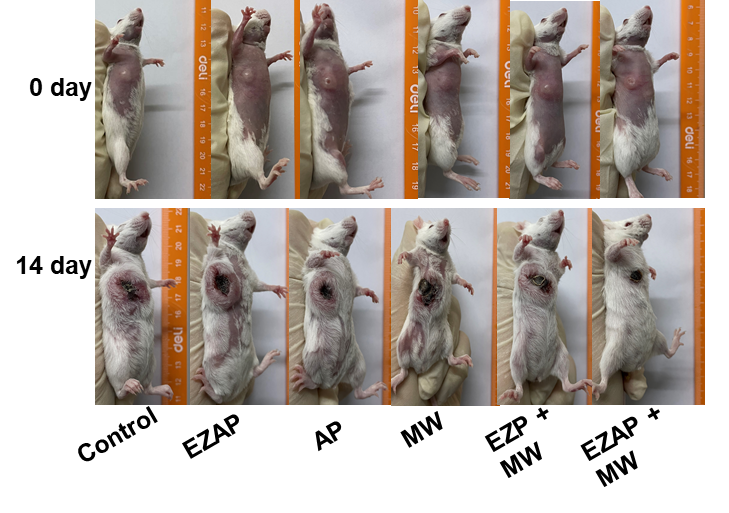


Figure S28. Representative images of different groups of tumor-bearing mice at 0 and 14 days respectively.

Figure S29. Emission spectra of EuMOF@ZIF-PEG at different excitation wavelengths.

Figure S30. The diagram between the maximum emission peak intensity and the excitation wavelength of EuMOF@ZIF-PEG.
